# 2,5-bis(4-alkoxycarbonylphenyl)-1,4-diaryl-1,4-dihydropyrrolo[3,2-*b*]pyrrole (**AAPP**) AIEgens: tunable RIR and TICT characteristics and their multifunctional applications†
†Electronic supplementary information (ESI) available: Selected spectra and data referred to in the paper. CCDC **AAPP** (1561488) and **AAPP-CF3** (1561489). For ESI and crystallographic data in CIF or other electronic format see DOI: 10.1039/c7sc03076b



**DOI:** 10.1039/c7sc03076b

**Published:** 2017-08-31

**Authors:** Kai Li, Yuanyuan Liu, Yuanyuan Li, Qi Feng, Hongwei Hou, Ben Zhong Tang

**Affiliations:** a College of Chemistry and Molecular Engineering , Zhengzhou University , Henan 450001 , P. R. China . Email: likai@zzu.edu.cn ; Email: houhongw@zzu.edu.cn; b College of Chemistry , Chemical and Environmental Engineering , Henan University of Technology , Henan 450001 , P. R. China; c Department of Chemistry , Hong Kong Branch of Chinese National Engineering Research Center for Tissue Restoration and Reconstruction , The Hong Kong University of Science & Technology , Clear Water Bay , Kowloon , Hong Kong , China . Email: tangbenz@ust.hk

## Abstract

Aggregation-induced emission luminogens (AIEgens) have attracted extensive interest for their outstanding luminescence properties in the aggregated state and even in the solid state. In this work, we developed a series of novel AIEgens based on 2,5-bis(4-alkoxycarbonylphenyl)-1,4-diaryl-1,4-dihydropyrrolo[3,2-*b*]pyrrole (**AAPP**). The AIEgens can be facilely synthesized through a single-step reaction under mild conditions with satisfactory yields. Interestingly, **AAPP** was found to have multiple luminous mechanisms that result in variable fluorescence properties. The propeller-like structure of **AAPP** enables a restricted intramolecular rotation (RIR) process which significantly enhances its fluorescence in the aggregated state (*i.e.* AIE fluorescence). In addition, there is a donor–acceptor interaction between the heterocycle center and the alkoxycarbonyl units in **AAPP** which allows a typical twisted intramolecular charge transfer (TICT) process in the dispersed state, resulting in strong fluorescence emissions in non-polar or low-polarity solvents but fluorescence quenching in high-polarity solvents. Due to the tunable RIR and TICT processes and the multiple fluorescence, **AAPP** compounds exhibit multifunctional applications: (1) as a reversible fluorescent thermometer, **AAPP** exhibited excellent fatigue resistance. There was a good linear relationship between its fluorescence intensity and temperature from 10 °C to 60 °C. (2) The desethyl **AAPP** derivative (**CAPP**) was successfully applied in the detection of Cd(ii) in aqueous solution at neutral pH, and showed a 500-fold fluorescence “turn-on” response to Cd(ii) with good selectivity.

## Abstract 2

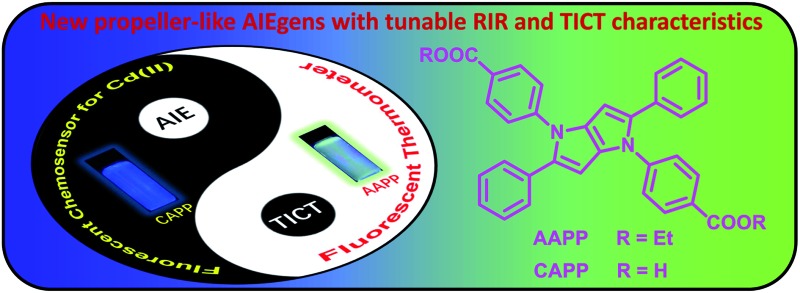
Novel propeller-like AIEgens with tunable emission were readily prepared and used as a fluorescent thermometer and selective chemosensor for Cd(ii) detection.

## Introduction

Aggregation-induced emission luminogens (AIEgens) are unique compounds with strong luminescence in the aggregated state and in the solid state.^1,2^ They have attracted great attention for their applications in materials science and biotechnology, including as organic light-emitting diodes, bioprobes/sensors, multistimuli-responsive materials, *etc.* ^3–10^ It has been demonstrated that the unique luminescence properties of most AIEgens originate from the restricted intramolecular rotation (RIR) mechanism.^11–13^ In dilute solution, AIEgens exist in a single molecule state. Their conjugate planes usually undergo dynamic intramolecular rotations in this case, which will cause the “excited state non-radiative transition” and result in fluorescence quenching. On the other hand, AIEgens can aggregate in concentrated solution (the aggregated state) and in the solid state. Under these circumstances, their intramolecular rotations are restricted, which inhibits the “excited state non-radiative transition” and makes the radiative transition a primary pathway for the electrons’ transition from the excited state to the ground state. Subsequently, fluorescence emission could be detected. This process is very different from that of traditional luminogens, whose fluorescence is usually quenched by π–π stacking in the aggregated state.^1^

To obtain compounds with good AIE properties a propeller-like structure is the most preferred type in the design of AIEgens as it can effectively avoid the fluorescence quenching caused by π–π stacking in the aggregated state.^14,15^ To date, a series of propeller-like AIEgens with excellent luminescence properties in the aggregated state have been developed, including hexaphenylsilole (HPS), 1,1-dimethyl-2,3,4,5-tetraphenylsilole (DMTPS), tetraphenylethene (TPE), tetraphenylpyrazine (TPP), tetraphenyl-1,4-butadiene (TPBD), *etc.* ^16–19^ Nevertheless, some intrinsic drawbacks of these reported AIEgens, which limit their potential applications, cannot be ignored. For example, a few of the classic propeller-like AIEgens (*e.g.* TPE and TPBD) contain double bonds, which usually give rise to *E*/*Z* isomerization and cause difficulty in the separation of the derivatives. The double bonds in these AIEgens may also lead to potential photobleaching or photooxidation which will result in a fluorescence loss.^20,21^ As well as these drawbacks in AIEgens with double bonds, some shortcomings also exist in other propeller-like AIEgens. For example, silole and its derivatives, HPS and DMTPS, are usually hard to prepare and are unstable under alkaline conditions.^17,22^ The core structure and the branching conjugation group determine the properties of AIEgens, which are so far still very limited in number and type. The development of AIEgens with a facile synthesis process, good stability and excellent luminescence properties remains challenging and in great need.

**Scheme 1:**
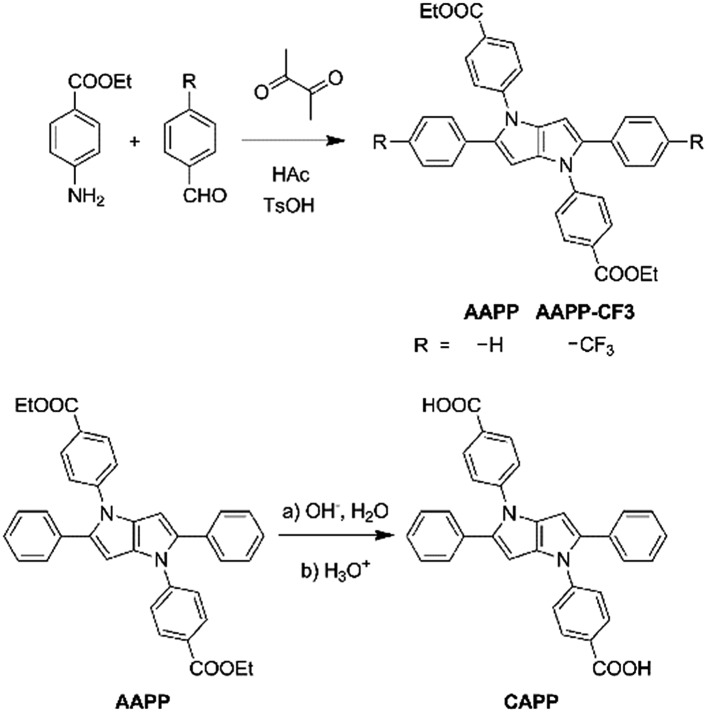
Synthesis of **AAPP**, **AAPP-CF3** and **CAPP**.

In this work, new propeller-like AIEgens of 2,5-bis(4-alkoxycarbonylphenyl)-1,4-diaryl-1,4-dihydropyrrolo[3,2-*b*]pyrrole (**AAPP**) and its derivatives are reported (Scheme 1). The compounds were facilely synthesized through single-step reactions under mild conditions with satisfactory yields. Interesting luminescence properties of **AAPP** were obtained. On the one hand, the propeller-like structure endowed **AAPP** and its derivatives with typical AIE properties due to the RIR process. On the other hand, there is a rotatable electron donor–accepter (D–A) structure in **AAPP**, which in addition gives it different luminescence properties in different solvents due to a twisted intramolecular charge transfer (TICT) process.^23,24^ When **AAPP** was dispersed in a high-polarity solvent, its fluorescence could be quenched completely. In contrast, **AAPP** exhibited a strong hypsochromic fluorescence emission in a non-polar solvent. In other words, the emission of **AAPP** originating from the TICT process is dependent on the polarity of the solvent. This property of polarity-dependent emission in solvents allowed **AAPP** to be applied as a reversible fluorescent thermometer in solvents (such as THF) when their polarity is proportional to temperature. As a fluorescent thermometer, **AAPP** showed excellent fatigue resistance in the temperature range from 10 °C to 60 °C in THF. Interestingly, when the ester group in **AAPP** was removed by a hydrolysis reaction, the AIE property of the desethyl-**AAPP** derivative (2,5-bis(4-carboxylphenyl)-1,4-diaryl-1,4-dihydropyrrolo[3,2-*b*]pyrrole, **CAPP**) almost disappeared, while its TICT property was still observed. **CAPP** exhibited a good solubility without fluorescence emission in aqueous solution. However, after the addition of Cd(ii), an over 500-fold fluorescence enhancement was observed, which was attributed to the formation of the chelate polymer of **CAPP-Cd**. Therefore, **CAPP** can be used as a “turn-on” fluorescence chemosensor for the detection of Cd(ii), which is one of the most poisonous metal ions to the human body.^25,26^ More importantly, **CAPP** exhibited a good selectivity for Cd(ii) over other metal ions with a satisfactory detection limit of 0.88 μmol L^–1^.

## Results and discussion

### Synthetic procedures

**AAPP** and its derivatives were readily synthesized with good yields by the coupling of ethyl-4-aminobenzoate and the corresponding benzaldehyde in glacial acetic acid.^27^ **CAPP** was obtained through a hydrolysis reaction of **AAPP** (Scheme 1). All of these compounds were characterized by ^1^H and ^13^C NMR spectroscopy and high-resolution mass spectroscopy (HRMS). Detailed synthetic procedures and characterization data are shown in the ESI.†

### Fluorescence characteristics of **AAPP**

**Fig. 1:**
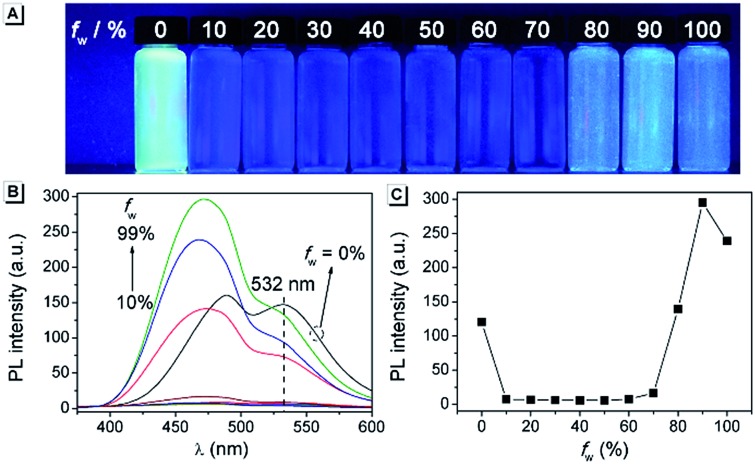
(A) Fluorescence photographs and (B) fluorescence emission spectra of **AAPP** in water/THF mixtures with different *f*_w_. (C) Fluorescence intensity of **AAPP** at 468 nm as a function of *f*_w_. Conditions: the concentrations of **AAPP** were 10 μmol L^–1^. The excitation wavelength was 322 nm. The photographs were taken under irradiation by 365 nm UV light.

The fluorescence characteristics of **AAPP** were first investigated in mixed solvents of water/THF (water fraction (*f*_w_) from 0% to 99%, v/v). As shown in Fig. 1A, **AAPP** exhibited strong green fluorescence in pure THF. However, it showed weak fluorescence in water/THF mixtures with *f*_w_ lower than 80%. When *f*_w_ was above 80%, blue fluorescence could be observed. The fluorescence color in the solvents with a high water fraction was apparently different from that in pure THF. In order to understand the emission differences, a series of experiments were carried out.

The fluorescence of **AAPP** in the mixed solvent of water/THF with a high water fraction was investigated first. As shown in Fig. 1B, the fluorescence intensity of **AAPP** at 468 nm in 99% water/THF was 42-fold higher than that of **AAPP** in 10% water/THF. Normally, water is a poor solvent for most organic compounds. Thus, the enhancement of the blue fluorescence with the increase in *f*_w_ might be due to the aggregation of **AAPP** in a poor solvent, *i.e.* an AIE fluorescence. To further verify that the blue emission in the water/THF mixtures originated from the aggregation of the **AAPP** molecules, absorption spectra were obtained (Fig. S1†). In pure THF, **AAPP** showed a strong absorption at 320 nm. However, with an increase in *f*_w_ the fine structures of the absorption spectra disappeared and level-off tails could be clearly observed in the visible region, which were believed to be due to the light scattering by the aggregated suspensions.^28,29^ As well as the absorption spectra, more direct evidence for the aggregation of **AAPP** in poor solvents was obtained from dynamic light scattering (DLS) measurements. As shown in the inset of Fig. S1,† particles of around 200 nm were detected in the 99% water/THF mixture. On the contrary, there were no particles observed in the pure THF solution. These results indicate that **AAPP** is fully soluble in THF but can form aggregates in poor solvents like water, which verified that the blue fluorescence of **AAPP** in the water/THF mixtures with high *f*_w_ was AIE-active.

**Fig. 2:**
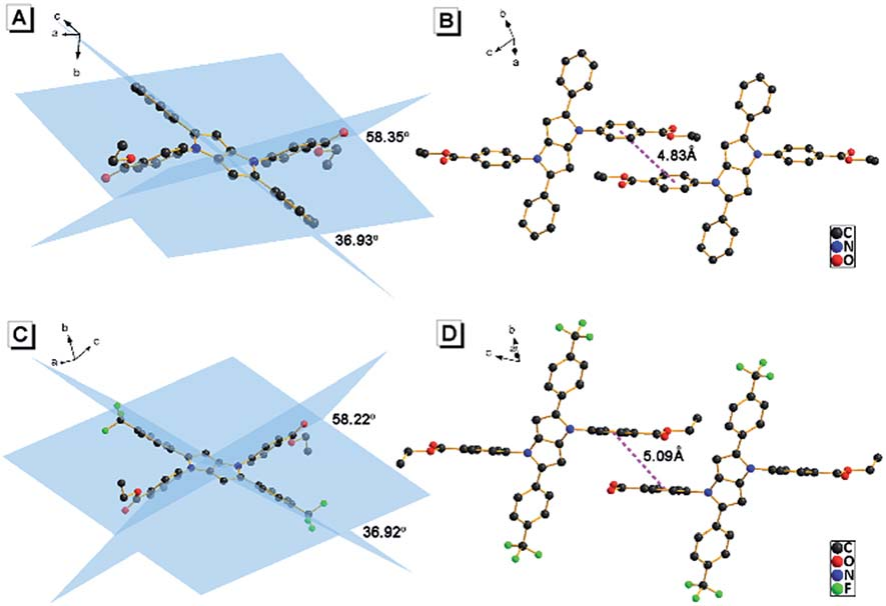
(A and B) Crystal structure of **AAPP**. (C and D) Crystal structure of **AAPP-CF3**. (A and C) show the dihedral angles between the aromatic rings and (B and D) show the minimum distance between the adjacent conjugate planes.

To understand the origin of the AIE character of **AAPP**, its crystal structure was further investigated. A single crystal of **AAPP** was grown from a methanol/THF mixture by a slow solvent evaporation method and was then characterized by X-ray crystallography. As shown in Fig. 2A, the dihedral angles between the aromatic rotors and the dihydropyrrolo[3,2-*b*]pyrrole center were 35.93° and 58.35°, respectively, which suggested that **AAPP** exhibited a typical propeller-like structure in the solid state. This propeller-like structure is beneficial to the RIR process in the aggregated state and could effectively weaken the π–π stacking interaction to avoid fluorescence quenching. The minimum distance between the adjacent conjugate planes was 4.83 Å (Fig. 2B), which is not conducive to the π–π stacking interaction in the crystal (normally, when the distance between the adjacent conjugate planes is larger than 3.8 Å, the π–π stacking interaction can be ignored^30^). Thus, the propeller-like structure without π–π stacking interactions in **AAPP** suggested that its AIE character originated from the RIR process. This explanation was further supported by the fluorescence spectra of **AAPP** in solvents with high viscosities (glycerin/ethanol). As shown in Fig. S2,† the fluorescence emission of **AAPP** was enhanced gradually with the increase in glycerin fraction (*f*_g_). This fluorescence enhancement was also due to the fact that the intramolecular rotations of **AAPP** were restricted by solvents with high viscosities. Therefore, the RIR process was determined to be the main cause of the AIE of **AAPP**.

As shown in Fig. 1, apart from the blue fluorescence in solvents with a high water fraction, a green fluorescence of **AAPP** was also observed in pure THF, which is quite unusual in most of the conventional propeller-like AIEgens. In order to identify the cause of the green emission, the fluorescence spectrum of **AAPP** in THF was measured. As shown in Fig. 1B (black line), the spectrum contained two emission peaks at 488 nm and 532 nm, which had nearly equal intensities. For the convenience of the following discussion, the shorter emission channel (488 nm) was named Em I while the longer emission channel (532 nm) was named Em II.

**Fig. 3:**
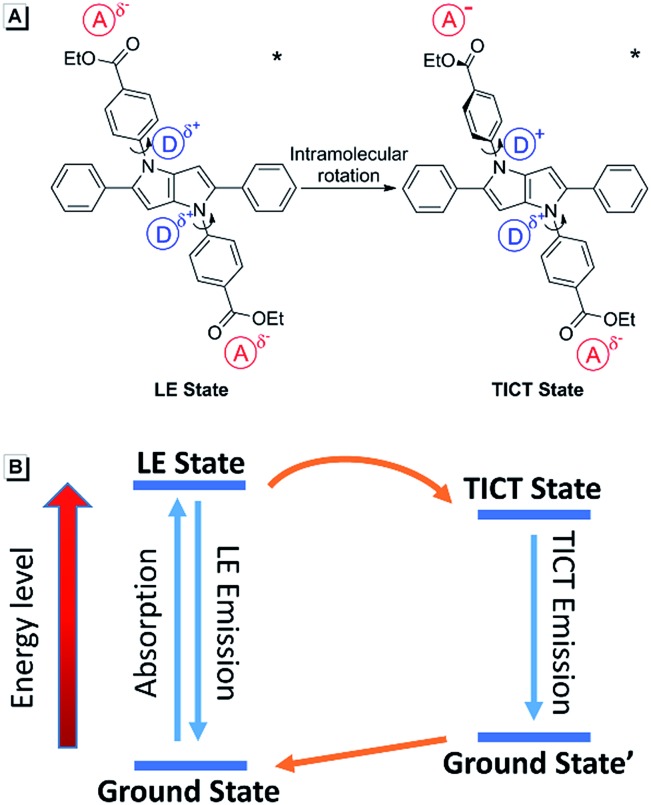
(A) Transition from the LE state of **AAPP** to the TICT state through intramolecular rotation of its donor and acceptor units at the excited state. (B) Energy diagram of the TICT process.

It was noticed that there are both typical electron donor (pyrrol-1-yl) and electron accepter (alkoxycarbonyl) structures in **AAPP**, which are connected by *para*-phenylene with rotatable single bonds (Fig. 3A). The existence of this typical rotatable D–A structure suggested that the fluorescence of **AAPP** in pure THF might originate from another luminous mechanism other than the RIR process, which is defined as the TICT process.^23,24^ According to the reports, luminogens with a rotatable D–A structure usually possess two excited states: a locally excited (LE) state and a TICT state (Fig. 3B).^31,32^ The LE state is the excited singlet state produced by light absorption, which is in equilibrium with solvent molecules in non-polar solvents. In this state, the donor and acceptor moieties in the excited luminogen molecules are coplanar. When going back to the ground state, a luminogen in the LE state usually produces a sharp and strong emission. The LE state can transform to a TICT state by intramolecular rotation of the donor and acceptor units in polar solvents. In the TICT state, the donor and acceptor moieties in the luminogen are more twisted and there is a charge separation between them. This charge separation in the D–A structure narrows the band gap and results in a bathochromic shift in the fluorescence spectra. Meanwhile, since the intramolecular rotation of its donor and acceptor units from the LE state to the TICT state is rather variable, there are different twisting angles in different luminogen molecules in the TICT state, which induce diverse emission characteristics for each molecule. As a whole, a broad and weak emission spectrum is often observed in the TICT state.^33^ The twisted molecular conformation is preferable in polar solvents and can be stabilized by the solvating effect of the polar solvent. Thus, increasing the polarity of the solvent can always bring the luminogens from the LE state to the TICT state, resulting in a large bathochromic shift in the emission wavelength and a dramatic decrease in the emission efficiency.^34,35^ As shown in Fig. 1, a green fluorescence can be observed in THF but no fluorescence was found for **AAPP** in the water/THF mixtures (with the water fraction between 10% and 70%). This fluorescence quenching of **AAPP** in the water/THF mixtures might be attributed to the TICT process because water is a highly polar solvent and this will be discussed in detail later.

**Fig. 4:**
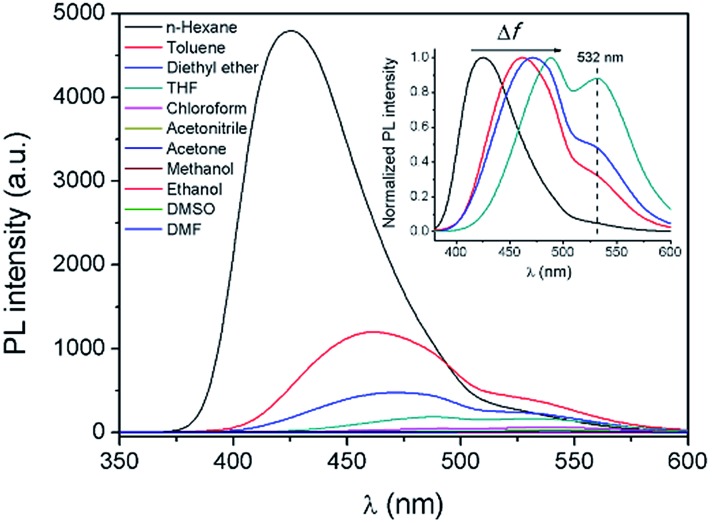
Fluorescence emission spectra of **AAPP** in different solvents. Inset: normalized fluorescence emission spectra of **AAPP** in solvents with low and medium polarity (from left to right: *n*-hexane, toluene, diethyl ether and THF). Conditions: the concentration of **AAPP** was 10 μmol L^–1^. The excitation wavelength was 322 nm.

In order to further explore the influence of the polarity of the solvent on the TICT process and the fluorescence properties, fluorescence spectra of **AAPP** in different solvents were investigated (Fig. 4). It is worth noting that the Em I intensity of **AAPP** was rather strong in the non-polar solvent *n*-hexane. However, the Em I intensity decreased dramatically in solvents with increased polarity. In highly polar solvents such as DMSO, acetonitrile, ethanol, *etc.*, the fluorescence of **AAPP** was too weak to be observed. In order to compare the fluorescence wavelengths more intuitively, the fluorescence spectra of **AAPP** in solvents with low and medium polarity were normalized and are shown in the inset of Fig. 4. It can be seen that the Em I wavelength of **AAPP** was bathochromically shifted from *n*-hexane to THF, which was in accordance with the bathochromic shift character of the TICT state in polar solvents as mentioned above. Therefore, based on the decreased Em I intensity and the bathochromic shift character in polar solvents, the Em I emission of **AAPP** was attributed to the TICT process originating from its rotatable D–A structure. The wavelength of Em II, which was different from that of Em I, however, was independent of the polarity of the solvent. As shown in Fig. 1B and the inset of Fig. 4, in all of the solvents the emission wavelength of Em II was around 532 nm, which indicated that the Em II emission was not derived from a TICT process. Instead, it was proposed to be due to the conjugation of the phenyl and dihydropyrrolo[3,2-*b*]pyrrole moieties.

**Fig. 5:**
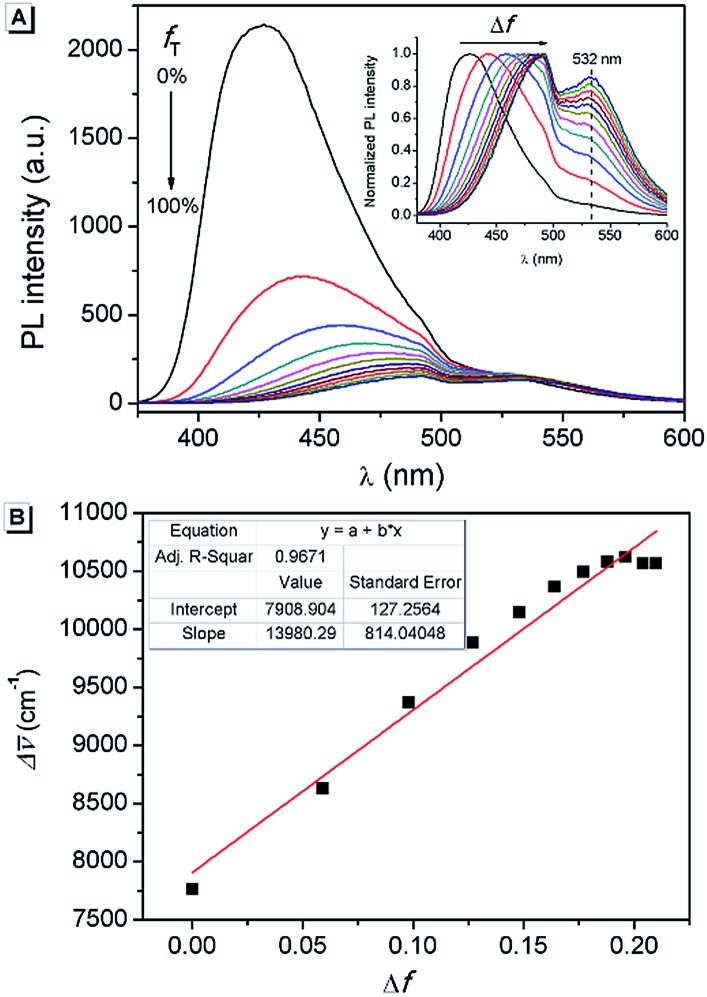
(A) Fluorescence emission spectra of **AAPP** in THF/*n*-hexane with different *f*_T_. Inset: normalized fluorescence emission spectra. (B) Lippert–Mataga plot of **AAPP**. Conditions: the concentration of **AAPP** was 10 μmol L^–1^. The excitation wavelength was 322 nm.

Furthermore, in order to understand the nature of the excited states associated with electronic transitions in **AAPP**, the absorption maxima (*λ*maxabs), fluorescence maxima (*λ*maxem), Stokes shift (Δῡ), fluorescence lifetime (*τ*) and quantum yield (*φ*) in mixed solvents of THF/*n*-hexane were measured. The results are shown in Fig. 5 and Table S1.† Using the equations *k*_r_ = *φ*/*τ* and *k*_nr_ = (1 – *φ*)/*τ* (where *k*_r_ and *k*_nr_ are the radiative decay rate constant and the non-radiative decay rate constant, respectively), the excited-state decay rate constants of **AAPP** could be estimated.^36^ As can be seen in Table S1,† *k*_nr_ was about 3 × 10^8^ s^–1^ in all of the mixtures of THF/*n*-hexane without apparent change. These results suggested that similar forms (the dispersed state) may be contained in all of the mixtures.^37^ In contrast, with the increase in the THF fraction (*f*_T_), *k*_r_ decreased gradually from 1.03 × 10^8^ s^–1^ to 4.22 × 10^6^ s^–1^, which implied that there might be different excited state charge distributions that are dependent on the polarity of the solvent.^38^ The solvent polarity function (Δ*f*) was calculated using eqn (1–3),^39^ in which the dielectric constants (*ε*) and refractive indices (*n*) of the pure solvents were taken from the literature.^40^ *f*_A_ and *f*_B_ in the equations are the fractions of the two solvents, respectively.

1 *ε*_MS_ = *f*_A_*ε*_A_ + *f*_B_*ε*_B_

2 *n*_MS_^2^ = *f*_A_*n*_A_^2^ + *f*_B_*n*_B_^2^

3
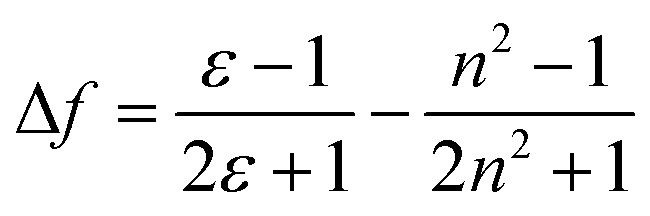



It was noticed that Δῡ of **AAPP** and Δ*f* of the solvent exhibit a good linear relationship for the mixtures of THF/*n*-hexane with different *f*_T_ (Fig. 5B), which can be described by the Lippert–Mataga plot:4
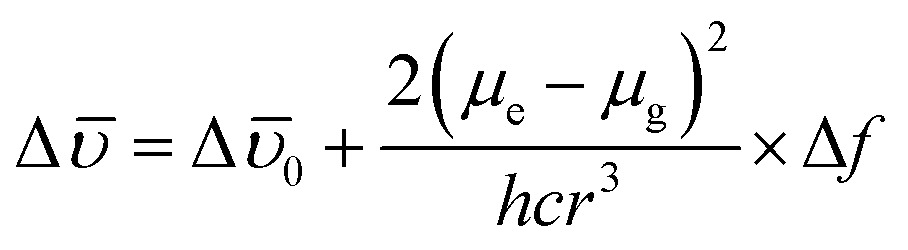
where *μ*_e_ and *μ*_g_ are the excited state dipole moment and the ground state dipole moment, respectively, *c* is the velocity of light, *h* is Planck’s constant, Δῡ_0_ is the Stokes shift of the fluorophore in a non-polar solvent and *r* is the radius of the Onsager cavity around the fluorophore.^41^ Since **AAPP** is a symmetrical molecule, *μ*_g_ is equal to 0. According to eqn (4), the linearity of the plot in Fig. 5B suggests that *μ*_e_ is not 0 (because (*μ*_e_ – *μ*_g_)^2^ > 0 and *μ*_g_ = 0), *i.e.* the excited state of **AAPP** shows a higher polarity than its ground state. The high polarity of the excited state indicates there was charge separation in the D–A structure, which agrees well with the TICT theory described in Fig. 3.

As has been studied above, **AAPP** could exhibit fluorescence not only from the RIR process (AIE) but also from the TICT process, depending on the solvent. In particular, a luminescence mechanism for **AAPP** in the water/THF solvent mixtures (Fig. 1) was concluded: in the solutions with a high water fraction, **AAPP** was in the aggregated state and the RIR process endowed it with a strong blue AIE fluorescence. In pure THF, **AAPP** was dissolved and exhibited two emissions of Em I and II. Em I was attributed to the emission in the TICT process that originated from the rotatable D–A structure while Em II originated from the conjugation of the phenyl and dihydropyrrolo[3,2-*b*]pyrrole moieties. In the solutions with a water fraction between 10% and 70%, **AAPP** was also dissolved. It was more twisted in the excited state because of the higher polarity of water than THF. There was no RIR process and the high polarity of the mixed solvents meant that **AAPP** was dominated by the TICT state, resulting in a quenching of the emission.

### Fluorescence characteristics of **AAPP-CF3**

To further understand the luminescence mechanism of **AAPP**, a typical electron-withdrawing group trifluoromethyl was introduced to the aryl moieties of **AAPP** to obtain the control compound **AAPP-CF3** (Scheme 1). On the one hand, **AAPP-CF3** was expected to exhibit AIE properties analogous to **AAPP** due to their similar propeller-like structures (Fig. 2). On the other hand, the trifluoromethyl group would balance the charge distribution in **AAPP-CF3** and weaken its TICT character.

As shown in Fig. S3,† an apparent fluorescence enhancement at 440 nm can be observed when the water fraction (*f*_w_) is above 60%, which indicates an AIE character of **AAPP-CF3**. In addition, with an increase in *f*_w_ the fine structures of the absorption spectra of **AAPP-CF3** disappeared and level-off tails could be observed in the visible region (Fig. S4†), which suggested the generation of aggregated suspensions. DLS measurement showed that the particle size of **AAPP-CF3** in the 99% aqueous solution was around 200 nm, which also proved the aggregation of **AAPP-CF3** in solvents with a high water fraction (Fig. S5†). In high viscosity solvents, **AAPP-CF3** also exhibited fluorescence enhancement like **AAPP** (Fig. S6†), which was due to the RIR process. All of these experiments indicated that **AAPP-CF3** exhibited a similar AIE character to **AAPP**.

**Fig. 6:**
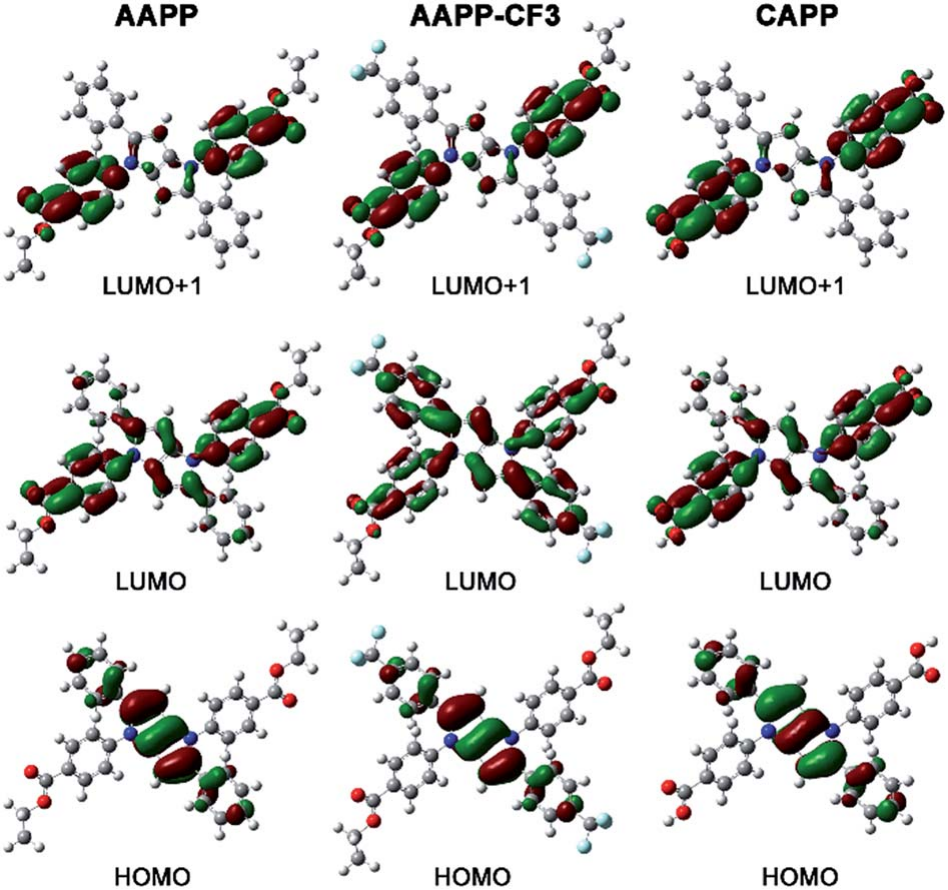
The Frontier molecular orbitals of **AAPP**, **AAPP-CF3** and **CAPP** obtained *via* DFT calculations. The geometries were optimized with the M06-2X density functional and the 6-31G (d,p) basis set. The value of the contour envelopes is 0.03 au.

The charge distributions of **AAPP** and **AAPP-CF3** were investigated by density functional theory (DFT) calculations. As reported, the photoexcitation of luminogens from the S0 to S1 state mainly involves electron transitions from the highest occupied molecular orbital (HOMO) to both the lowest unoccupied molecular orbital (LUMO) and the LUMO+1.^42–44^ For TICT luminogens, the HOMO is usually located on their electron donor moieties while the LUMO and LUMO+1 are located on their electron accepter moieties.^45–47^ Therefore, there will be a strong charge transfer process from the HOMO to the LUMO and LUMO+1 in the excited states, which is also a typical characteristic of TICT luminogens. As shown in Fig. 6, the HOMO of **AAPP** is mainly located on the phenyl and central heterocycle moieties (electron donors) while the LUMO and LUMO+1 are mostly positioned on the 4-ethoxycarbonylphenyl moieties (electron accepters). These distinct charge distributions in **AAPP** enable it to undergo a typical TICT process, which is in accordance with reported TICT luminogens.^45–47^ In contrast, the charge distribution of **AAPP-CF3** is more balanced than that of **AAPP**. As shown, the HOMO and LUMO+1 of **AAPP-CF3** are analogous to those of **AAPP**. However, the LUMO of **AAPP-CF3** is located on the whole molecule, which is very different from that of **AAPP**. These balanced charge distributions of **AAPP-CF3** suggest that there should be a weaker TICT character for **AAPP-CF3** than **AAPP**, which will be shown by a shorter wavelength and stronger fluorescence emission in **AAPP-CF3** as compared with **AAPP**.

**Fig. 7:**
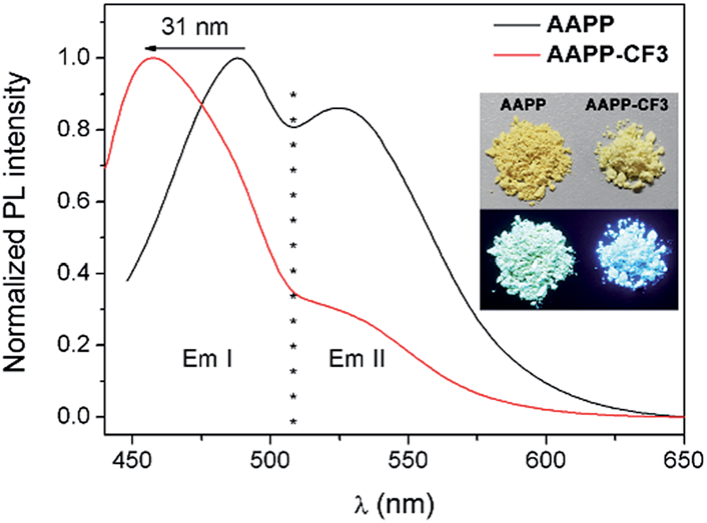
Fluorescence emission spectra of **AAPP** and **AAPP-CF3** in the solid state. Inset: photographs of **AAPP** and **AAPP-CF3** in the solid state excited by sunlight (upper) and 365 nm UV light (lower).

In order to clarify the weaker TICT character of **AAPP-CF3** than **AAPP**, the luminescence properties of **AAPP-CF3** in both solution and in the solid state were investigated. As shown in Fig. S7 and Table S2,† the Em I wavelengths of **AAPP-CF3** in *n*-hexane and THF were 417 nm and 430 nm, respectively, and were both shorter than those of **AAPP** under the same conditions (424 nm and 488 nm, respectively). Meanwhile, the quantum yield of **AAPP-CF3** was 77.95% in *n*-hexane and 26.41% in THF, which were also much higher than those of **AAPP** (26.68% and 1.24%, respectively). Though there was still a bathochromic shift in the Em I wavelength and a decrease in the Em I efficiency for **AAPP-CF3** with the increasing polarity of the solvent (Fig. S7 and S8†), the variation ranges were much smaller than those of **AAPP**. As shown in Fig. 7, the Em I wavelength of **AAPP-CF3** was 31 nm shorter than that of **AAPP** in the solid state. Meanwhile, the fluorescence quantum yield of **AAPP-CF3** was 17.89%, which was also higher than that of **AAPP** (8.72%). All of these results suggest that the TICT character of **AAPP-CF3** is weaker than that of **AAPP**. Interestingly, the Em II wavelength of **AAPP-CF3** was very close to that of **AAPP** (Fig. 7), which indicated their analogous conjugated structures (the trifluoromethyl groups are not conjugated with the phenyl moieties). These results again prove that Em II of **AAPP** does not originate from a TICT process but is attributed to the conjugation of the phenyl and dihydropyrrolo[3,2-*b*]pyrrole moieties.

### The application of **AAPP** as a fluorescent thermometer

**Fig. 8:**
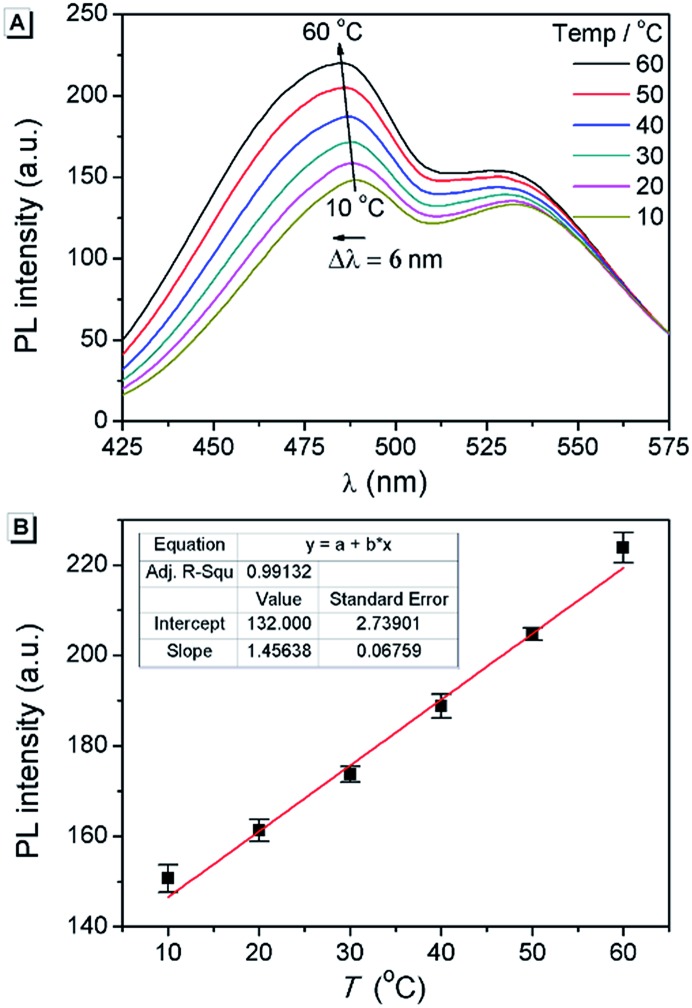
(A) Temperature dependence of the fluorescence spectra of **AAPP** in THF. (B) Temperature dependence of the fluorescence intensity of **AAPP** in THF at 488 nm and the associated best-fit equations. Conditions: the concentration of **AAPP** is 10 μmol L^–1^. The excitation wavelength was 322 nm.

Fluorescent thermometers are very useful in chemistry and biology.^48^ It is known that TICT luminogens are sensitive to temperature because of the thermally activated and solvent polarity-assisted intramolecular interconversion of the emitting states.^23,49^ As shown in Fig. 8A, the fluorescence intensity of **AAPP** in THF increased gradually with the increase in temperature from 10 °C to 60 °C. Meanwhile, a hypsochromic shift of the fluorescence maximum could be also observed, which was due to the decreased polarity of THF when the temperature increased. As shown in Fig. 8B, the fluorescence intensity at 488 nm increased linearly with the increasing temperature, which afforded an excellent positive linear correlation with a correlation coefficient *R* ^2^ = 0.991 (*n* = 5). More importantly, as a reversible fluorescent thermometer, the fatigue resistance of **AAPP** was very good. It can be seen from Fig. S9† that even though **AAPP** was toggled repeatedly between the two temperatures of 10 °C and 60 °C in THF 10 times, the fluorescence intensity at 488 nm remained almost constant without any apparent degradation. This excellent fatigue resistance of **AAPP** indicated that its stability was also very good. Thus, **AAPP** could be used as a reversible fluorescent thermometer with a positive temperature coefficient.

### Fluorescence characteristics of **CAPP** and its application in the detection of Cd(ii)

When the ethyl group of **AAPP** was removed by a hydrolysis reaction, the new compound of 2,5-bis(4-carbonylphenyl)-1,4-diaryl-1,4-dihydropyrrolo[3,2-*b*]pyrrole (**CAPP**) was obtained (Scheme 1). As shown in Fig. 6, the charge distribution of **CAPP** is similar to that of **AAPP** due to their analogous rotatable D–A structures. These similarities suggest that **CAPP** may exhibit a similar TICT fluorescence to **AAPP**. As expected, the Em I intensity of **CAPP** decreased and the wavelength bathochromically shifted with the increasing solvent polarity, indicating a typical TICT process (Fig. S10†). Meanwhile, the Em II wavelength of **CAPP** was independent of the polarity of the solvent (Fig. S10† inset), which was also analogous to **AAPP**. However, **CAPP** exhibited no fluorescence in aqueous solution, which was very different to **AAPP**. This may be due to the good water solubility of **CAPP**, which makes it completely soluble in aqueous solution and thus it does not aggregate and exhibits no AIE fluorescence.

**Fig. 9:**
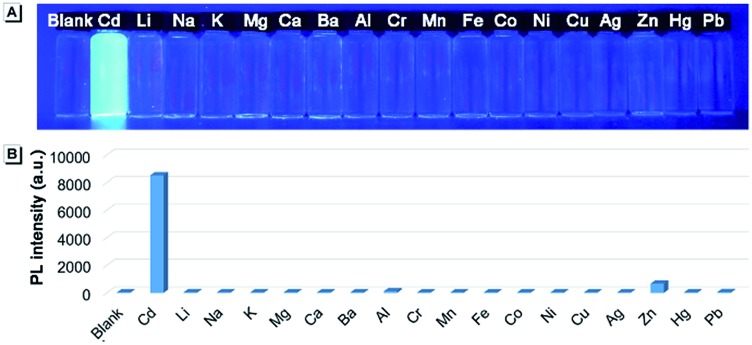
(A) Photograph and (B) fluorescence intensities of **CAPP** at 445 nm in the presence of different metal ions. Ions from left to right are blank, Cd(ii), Li(i), Na(i), K(i), Mg(ii), Ca(ii), Ba(ii), Al(iii), Cr(iii), Mn(ii), Fe(iii), Co(ii), Ni(ii), Cu(ii), Ag(i), Zn(ii), Hg(ii) and Pb(ii). Conditions: 90% water/THF (v/v) at pH 7.0 controlled by a 10 mmol L^–1^ Tris buffer solution and the concentrations of **CAPP** and metal ions were 10 μmol L^–1^. The photograph was taken under irradiation by 365 nm UV light.

We wondered if there was any method to regain the fluorescence of **CAPP** in aqueous solution. According to previous reports, carboxyl-containing AIEgens usually exhibit a significant fluorescence enhancement after binding with certain metal ions due to the formation of chelate polymers.^50–52^ The aggregation of AIEgens in chelate polymers would lead to an RIR process which is beneficial to their AIE fluorescence. As shown in Fig. 9, different metal ions were added to an aqueous solution of **CAPP** to investigate their influence on the fluorescence properties. Interestingly, only Cd(ii) can lead to a significant fluorescence “turn-on” response. Even in the presence of Zn(ii), which usually induces comparable fluorescence responses to that of Cd(ii),^26,53^ the fluorescence enhancement was almost negligible. These results suggested that **CAPP** exhibited an excellent selectivity and might be used as a chemosensor for Cd(ii) detection.

**Fig. 10:**
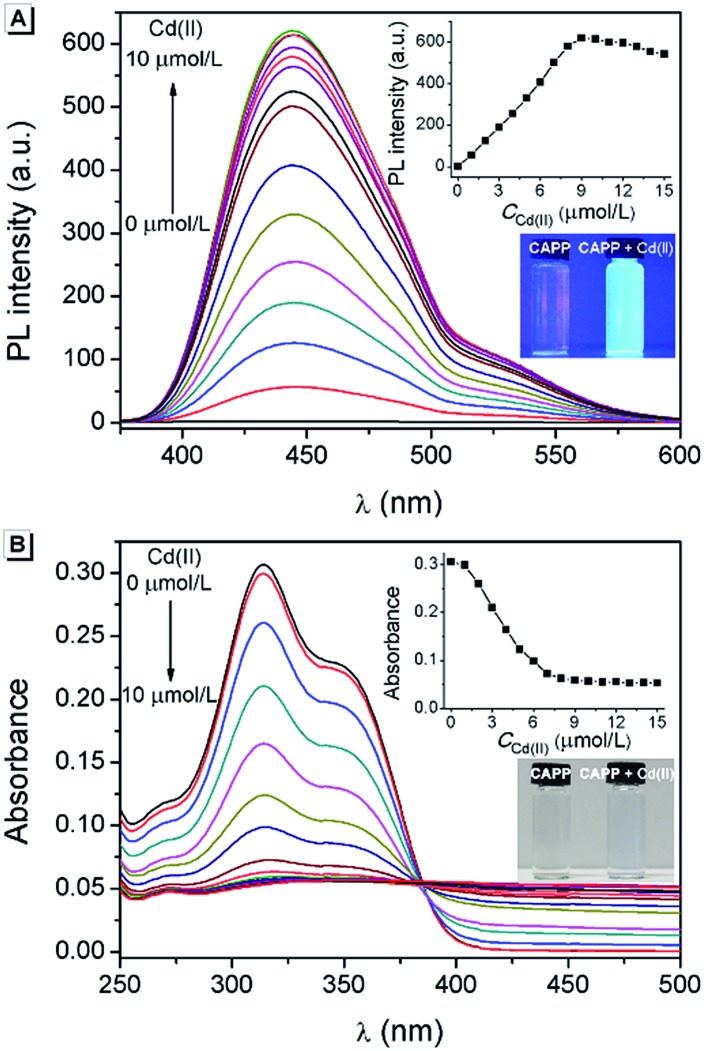
(A) Fluorescence spectra and (B) absorption spectra of 10 μmol L^–1^ **CAPP** upon the addition of Cd(ii). Inset in (A): the fluorescence intensity at 445 nm as a function of Cd(ii) concentration (excitation was at 360 nm). Inset in (B): the absorbance at 314 nm as a function of Cd(ii) concentration. The photos are **CAPP** in the absence and presence of 1 equiv. Cd(ii) in a glass cuvette under irradiation by (A) 365 nm UV light and (B) sunlight. Conditions: 90% water/THF (v/v) at pH 7.0 controlled by a 10 mmol L^–1^ Tris buffer solution.

**Fig. 11:**
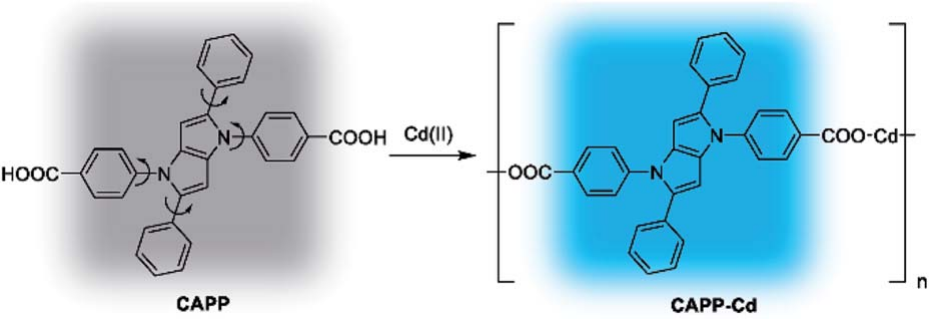
Proposed mechanism for the fluorescence enhancement of **CAPP** by Cd(ii).

Cadmium is widely used in electroplating, metallurgy, the production of batteries, *etc.* ^54^ However, it is one of the most poisonous metals to the human body and can cause osteoporosis, leukaemia and several cancers. The divalent cadmium ion (Cd(ii)) can enter the body mainly through food, water and air and is unfortunately difficult to biodegrade.^25,26,55–57^ It has been strictly defined by the World Health Organization (WHO) that the daily oral intake of Cd(ii) cannot exceed 35 μg.^58^ Therefore, the detection of Cd(ii) in environmental samples is essential and meaningful. For this reason, the performance of **CAPP** as a fluorescence chemosensor for Cd(ii) detection was further investigated in this work. As illustrated in Fig. 10A, a more than 500-fold fluorescence enhancement at 445 nm can be observed upon the addition of 1 equiv. Cd(ii) to 10 μmol L^–1^ **CAPP** in 90% water/THF (v/v) at pH 7.0. A proposed mechanism for the fluorescence enhancement of **CAPP** by Cd(ii) is shown in Fig. 11. The weak fluorescence emission of **CAPP** in aqueous solution was due to its intramolecular rotation in the dispersed state. After binding with Cd(ii), chelate polymers of **CAPP-Cd** were formed and the intramolecular rotation was restricted, resulting in an enhancement of the blue fluorescence.

To verify that the fluorescence enhancement was induced by aggregation, the absorption spectra of **CAPP** in the presence of different concentrations of Cd(ii) were recorded. As shown in Fig. 10B, **CAPP** displayed a structured absorption spectrum with a maximum absorption at 314 nm in the absence of Cd(ii). After the addition of Cd(ii), the fine structures of the absorption spectra disappeared gradually and level-off tails were observed in the visible region, indicating the formation of aggregation. In addition, particles of around 1000 nm were detected in the presence of 1 equiv. Cd(ii) by DLS measurement, which provides more direct evidence for aggregation (Fig. S11†). The metal-to-ligand ratio in **CAPP-Cd** was investigated using Job’s plot method from the fluorescence spectra of **CAPP** and Cd(ii) with a total concentration of 20 μmol L^–1^ (Fig. S12†), and the results suggested the formation of a 1 : 1 complex. Time-dependent fluorescence spectra were further recorded to monitor the formation and decomposition processes of the **CAPP-Cd** aggregation (Fig. S13†). It can be seen that the fluorescence intensity at 445 nm was enhanced gradually after the addition of 1 equiv. Cd(ii) and reached its maximum within 10 min. When EDTA was added to the system, the fluorescence decreased slowly and dropped to its minimum after about 100 min. These gradual changes of fluorescence intensity were due to the fact that the formation and decomposition processes of chelate polymers usually require a longer reaction time than those of simple chelates, again verifying the generation of chelate polymers between **CAPP** and Cd(ii).

As a “turn-on” fluorescence chemosensor, the optimal pH for the detection of Cd(ii) using **CAPP** was investigated. As shown in Fig. S14,† the fluorescence of **CAPP** was very weak from pH 5.0 to pH 9.0. In contrast, after the addition of Cd(ii), the fluorescence enhanced dramatically at pH > 6.0 and reached a maximum at neutral pH. In acidic solutions, the protonation of the carboxyl group weakened the binding ability of **CAPP** to Cd(ii), which was unfavorable for the formation of **CAPP-Cd**. Whereas in alkaline solutions, the amount of **CAPP-Cd** decreased due to the competition between OH^–^ and **CAPP** in binding to Cd(ii). Thus, in order to achieve a higher signal-to-noise ratio, pH 7.0 was employed for Cd(ii) detection.

**Fig. 12:**
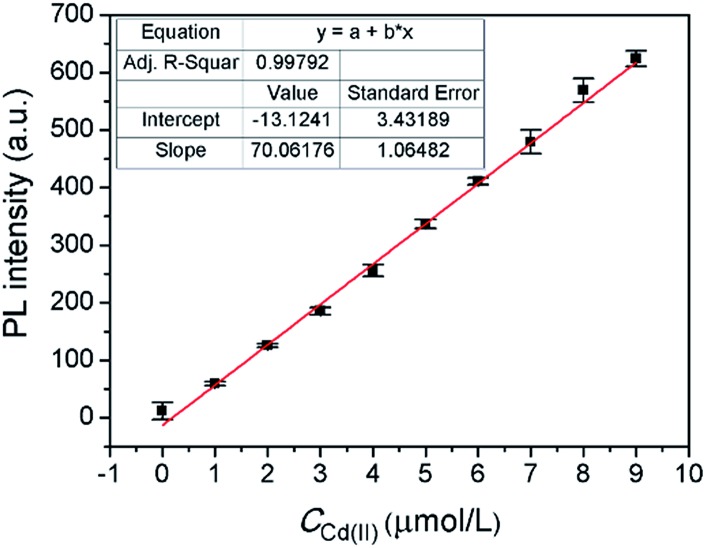
Fluorescence intensity at 445 nm of 10 μmol L^–1^ **CAPP** as a function of Cd(ii) concentration. Conditions: 90% water/THF (v/v) at pH 7.0 controlled by a 10 mmol L^–1^ Tris buffer solution. Excitation and emission was performed at 360 nm and 445 nm, respectively.

Under the optimal conditions, a satisfying analytical performance of **CAPP** was obtained in the detection of Cd(ii). As shown in Fig. 12, the calibration curve for the determination of Cd(ii) by **CAPP** was plotted, which showed that the linear range was at least 0.0–9.0 μmol L^–1^ with a correlation coefficient *R* ^2^ = 0.998 (*n* = 3). According to the definition by IUPAC (*C*_DL_ = 3*S*_b_/*m*, where *C*_DL_ is the detection limit, *S*_b_ is the standard deviation from 10 blank solutions and *m* represents the slope of the calibration curve^59^), the detection limit was calculated to be 0.88 μmol L^–1^.

## Conclusions

In conclusion, a new type of AIEgen of **AAPP** and its derivatives were developed, which can be facilely synthesized by a single-step reaction under mild conditions with satisfactory yields. The luminescence mechanism of **AAPP** in different solvents and in the solid state was studied. **AAPP** is a propeller-like molecule and exhibits typical AIE characteristic due to the RIR mechanism. Meanwhile, the rotatable D–A structure in **AAPP** makes it have different emissions in different solvents due to a TICT process. The RIR and TICT processes of **AAPP** are tunable by the solvent polarity, temperature and substituent groups, resulting in multiple luminescence properties. **AAPP** and its derivative **CAPP** have been successfully used in temperature sensing and Cd(ii) detection, respectively, and both exhibited a good performance. As a reversible fluorescent thermometer, **AAPP** exhibited excellent fatigue resistance. There was a good linear relationship between its fluorescence intensity and temperature from 10 °C to 60 °C. **CAPP** can be used as a “turn-on” fluorescence chemosensor, which exhibited a 500-fold fluorescence enhancement in response to Cd(ii) in aqueous solution at neutral pH. Meanwhile, the selectivity of **AAPP** for the detection of Cd(ii) was very good and a detection limit of 0.88 μmol L^–1^ was achieved. Currently, efforts toward developing more multifunctional molecules based on **AAPP** AIEgens are under investigation in our laboratories.

## Conflicts of interest

There are no conflicts to declare.
