# Supplementary material for: 2,5-bis(4-alkoxycarbonylphenyl)-1,4-diaryl-1,4-dihydropyrrolo[3,2-b]pyrrole (AAPP) AIEgens: tunable RIR and TICT characteristics and their multifunctional applications
Source: Chem Sci. 2017 Aug 31;8(10):7258–67. doi: 10.1039/c7sc03076b (PMC5633666; doi:10.1039/c7sc03076b)
Supplement: Supplementary file 1 [file SC-008-C7SC03076B-s001.pdf]

Electronic Supplementary Material (ESI) for Chemical Science.
This journal is © The Royal Society of Chemistry 2017

2,5-bis(4-alkoxycarbonylphenyl)-1,4-diaryl-1,4-dihydropyrrolo[3,2-b]pyrrole (AAPP) AIEgens: tunable RIR and TICT characteristics and their multifunctional applications

Kai Li,*[ac] Yuanyuan Liu,[a] Yuanyuan Li,[b] Qi Feng,[a] Hongwei Hou*[a] and Ben Zhong Tang*[c]

[a] College of Chemistry and Molecular Engineering, Zhengzhou University, Henan 450001, P. R. China.

[b] College of Chemistry, Chemical and Environmental Engineering, Henan University of Technology, Henan 450001, P. R. China.

[c] Department of Chemistry and Hong Kong Branch of Chinese National Engineering Research Center for Tissue Restoration and Reconstruction, The Hong Kong University of Science & Technology, Clear Water Bay, Kowloon, Hong Kong, China.

Contents

1. Experimental..S2

2. Synthesis..S3

3. Selected spectra and data referred in the paper..S4

4. NMR spectra and HRMS spectra...S11

5. Crystallographic data and structure refinement...S16

1. Experimental

1.1 Reagents

All commercially available reagents and solvents of analytical grade were used as received unless otherwise mentioned. Ethyl-4-aminobenzoate, benzaldehyde, 4-(trifluoromethyl)benzaldehyde and 2,3-butanedione were purchased from J&K Chemical Co., Beijing, China. All the other materials such as solvents and metal salts were purchased from Sinopharm Chemical Reagent Beijing Co., Beijing, China. All the metal ions used in the experiment were prepared from their nitrate salts or perchlorate salts. Deionized water (distilled) was used in the whole experiment. Tris buffer solutions were prepared using 10 mmol/L Tris and proper amount of nitric acid under adjustment by a pH meter. HAc/NaAc buffer solutions were prepared using 10 mmol/L NaAc and proper amount of nitric acid under adjustment by a pH meter. Unless otherwise noted, all of the absorption and fluorescence spectra were recorded at room temperature.

1.2 Apparatus

Fluorescence spectra were recorded on a JASCO-FP-8300 fluorescence spectrophotometer, 1 cm quartz cell. The temperatures in fluorescence measurements were controlled by an ETC-815 peltier thermostatted single cell holder, which offered a temperature control accuracy of ±0.1 ^{o}C. Absorption spectra were determined using a JASCO-750 UV-vis spectrophotometer, 1 cm quartz cell. Fluorescence quantum yields and fluorescence lifetimes were recorded on an Edinburgh FlS-980 fluorescence spectrometer. Dynamic light scattering (DLS) experiments were carried on a NanoPlus-3 DLS particle size/zeta potential analyzer. The pH was determined by a Mettler Toledo FE20/EL20 pH meter. All of the nuclear magnetic resonance (NMR) spectra were recorded on a Bruker 400 Avance NMR spectrometer operated at 400 MHz. High resolution mass spectra (HRMS) were obtained on a GCT premier CAB048 mass spectrometer operating in matrix assisted laser desorption ionization time-of-flight (MALDI-TOF) mode. Single-crystal X-ray diffraction intensity data were recorded using a Rigaku Saturn 724 CCD diffractometer with Mo Ka radiation

(λ = 0.71073 Å) at room temperature. The photos were taken by a Nikon D5500 camera.

2. Synthesis

2,5-bis(4-alkoxycarbonylphenyl)-1,4-diaryl-1,4-dihydropyrrolo[3,2-b]pyrrole (**AAPP**).

Ethyl-4-aminobenzoate (1.65 g, 10 mmol), benzaldehyde (1.06 g, 10 mmol) and p-toluenesulfonic acid (0.17 g, 1 mmol) were dissolved in 10 mL glacial acetic acid in a 25 mL flask. The mixture was heated to 90 ^{o}C and stirred for 1 h. Then 2,3-butanedione (0.43 g, 5 mmol) was added dropwise and the resulting mixture was stirred at 90 °C for 3 h to yield a yellow precipitate. After cooling to room temperature, the precipitate was filtered and washed by 15 mL glacial acetic acid for three times. The crude product was purified by recrystallization in THF to get 1.20 g **AAPP** (yield 43%) as yellow solid. ^{1}H NMR ($CDCl_3$) δ (ppm): 8.07 (d, 4H, J = 8.0 Hz), 7.35 (d, 4H, J = 8.0 Hz), 7.27 (m, 10H), 6.50 (s, 2H), 4.41 (q, 4H, J = 8.0 Hz), 1.42 (t, 6H, J = 8.0 Hz). ^{13}C NMR (DMSO-d_6) δ (ppm): 166.09, 143.69, 136.03, 133.18, 131.26, 128.43, 128.32, 127.46, 126.74, 124.37, 96.41, 61.06, 14.37. HRMS spectrometry: m/z calcd for $[C_{36}H_{30}N_2O_4]^+$: 554.2206; found: 554.2197.

2,5-bis(4-alkoxycarbonylphenyl)-1,4-bis(4-(trifluoromethyl)phenyl)-1,4-dihydropyrrolo[3,2-b]pyrrole (**AAPP-CF3**).

Ethyl-4-aminobenzoate (1.65 g, 10 mmol), 4-(trifluoromethyl)benzaldehyde (1.74 g, 10 mmol) and p-toluenesulfonic acid (0.17 g, 1 mmol) were dissolved in 10 mL glacial acetic acid in a 25 mL flask. The mixture was heated to 90 ^{o}C and stirred for 1 h. Then 2,3-butanedione (0.43 g, 5 mmol) was added dropwise and the resulting mixture was stirred at 90 °C for 3 h to yield a yellow precipitate. After cooling to room temperature, the precipitate was filtered and washed by 15 mL glacial acetic acid for three times. The crude product was purified by recrystallization in THF to get 1.27 g **AAPP-CF3** (yield 37%) as yellow solid. ^{1}H NMR ($CDCl_3$) δ (ppm): 8.09 (d, 4H, J = 8.0 Hz), 7.51 (d, 4H, J = 8.0 Hz), 7.32 (m, 8H), 6.54 (s, 2H), 4.38 (q, 4H, J =

8.0 Hz), 1.41 (t, 6H, J = 8.0 Hz). ^{13}C NMR (DMSO-d_6) δ (ppm): 165.87, 143.11, 136.37, 135.16, 132.26, 130.96, 128.66, 128.34, 128.10, 128.06, 125.49, 125.46, 125.42, 124.56, 122.79, 97.22, 61.22, 14.35. HRMS spectrometry: *m/z* calcd for $[C_{38}H_{28}F_6N_2O_4]^+$: 690.1953; found: 690.1970.

2,5-bis(4-carboxylphenyl)-1,4-diaryl-1,4-dihydropyrrolo[3,2-b]pyrrole (**CAPP**)

AAPP (1.11 g, 2 mmol) and NaOH (0.40 g, 10 mmol) were dissolved in 100 mL THF in a 250 mL flask. The mixture was heated to 90 °C and stirred for 3 h. Then excess HCl was added to adjust the pH of the mixture to acidity, yielding a yellow precipitate. The precipitate was filtered and dried under reduced pressure. The crude product was purified by recrystallization in DCM to get 0.96 g **CAPP** (yield 96%) as yellow solid. ^{1}H NMR (DMSO-d_6) δ (ppm): 13.03 (s, 2H), 7.98 (d, 4H, J = 12.0 Hz), 7.38 (d, 4H, J = 12.0 Hz), 7.31 (m, 4H), 7.24 (m, 6H), 6.64 (s, 2H). ^{13}C NMR (DMSO-d_6) δ (ppm): 167.20, 143.43, 135.80, 133.21, 131.53, 131.07, 128.97, 128.20, 127.14, 124.88, 97.29. HRMS spectrometry: *m/z* calcd for $[C_{32}H_{22}N_2O_4]^+$: 498.1580; found: 498.1601.

1. Selected spectra and data referred in the paper

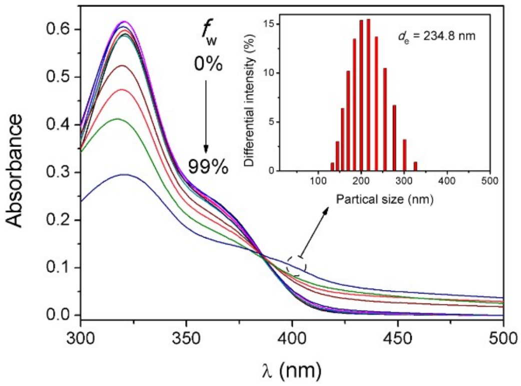

Figure S1. Absorption spectra of **AAPP** in water/THF mixtures with different f_w. Inset: DLS results of **AAPP** in aqueous solution of 99% water/THF (*v*/*v*). Conditions: The concentration of **AAPP** was 10 μmol/L.

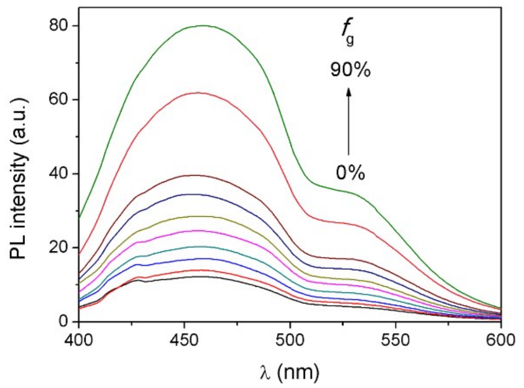

Figure S2. Fluorescence spectra of **AAPP** in glycerin/ethanol mixtures with the glycerin fraction (f_g) increased from 0% to 90%. Conditions: The concentration of **AAPP** was 10 μmol/L. The excitation wavelength was 322 nm.

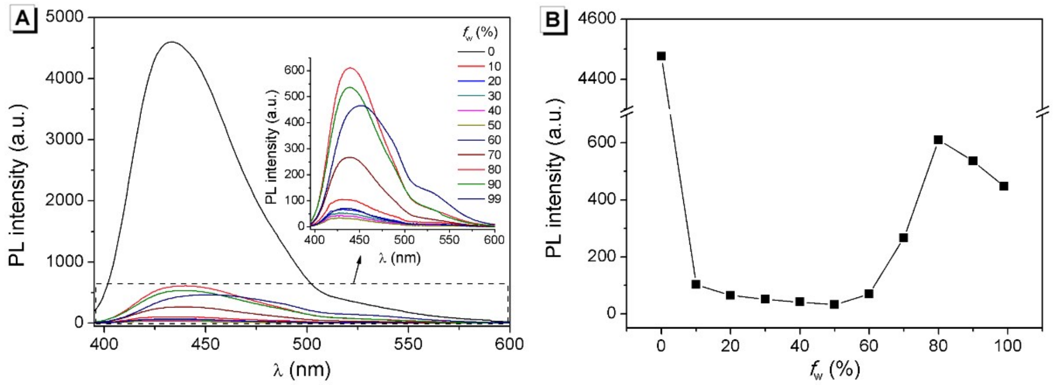

Figure S3. A) Fluorescence emission spectra of **AAPP-CF3** in water/THF mixtures with different f_w. B) Fluorescence intensity of **AAPP-CF3** at 440 nm as a function of f_w. Conditions: The concentration of **AAPP-CF3** was 10 μmol/L. The excitation wavelength was 326 nm.

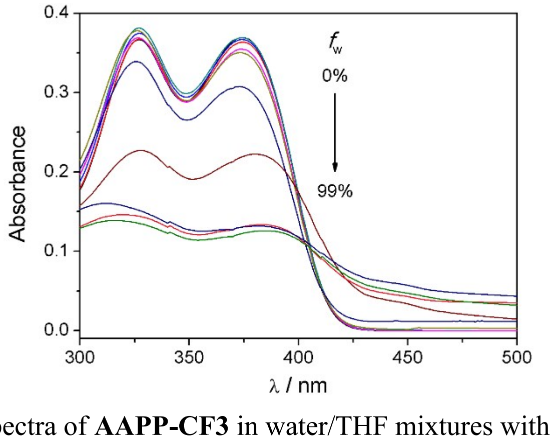

Figure S4. Absorption spectra of **AAPP-CF3** in water/THF mixtures with different f_w. Conditions: The concentration of **AAPP-CF3** was 10 μmol/L.

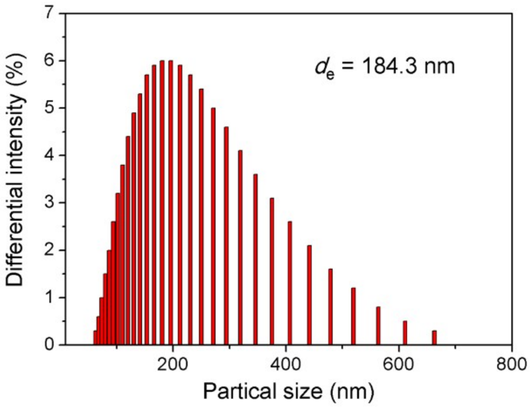

Figure S5. DLS results of **AAPP-CF3** in aqueous solution of 99% water/THF (*v*/*v*). Conditions: The concentration of **AAPP-CF3** was 10 μmol/L.

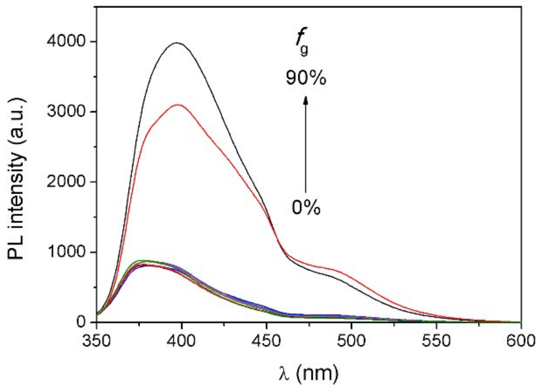

Figure S6. Fluorescence spectra of **AAPP-CF3** in glycerin/ethanol mixtures with the glycerin fraction (f_g) increased from 0% to 90%. Conditions: The concentration of **AAPP-CF3** was 10 μmol/L. The excitation wavelength was 326 nm.

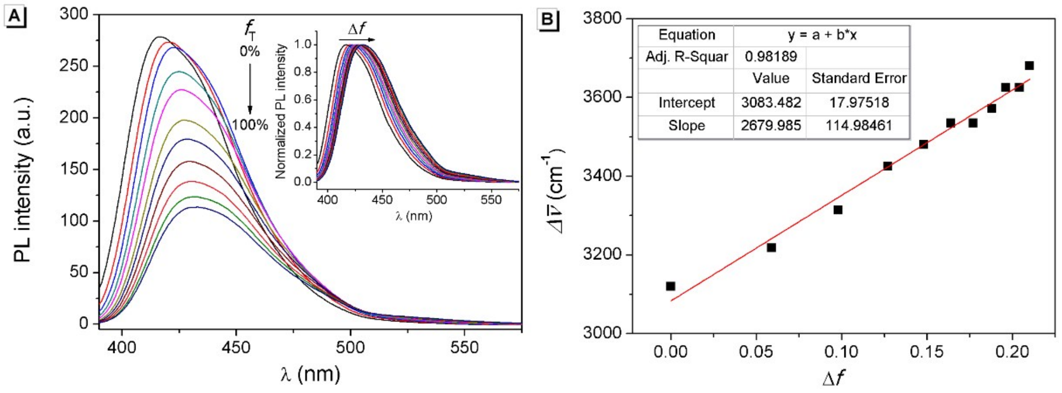

Figure S7. A) Fluorescence emission spectra of **AAPP-CF3** in THF/n-hexane with different f_T. Inset: Normalized fluorescence emission spectra. B) Lippert-Mataga plot of **AAPP-CF3**. Conditions: The concentration of **AAPP-CF3** was 10 μmol/L. The excitation wavelength was 326 nm.

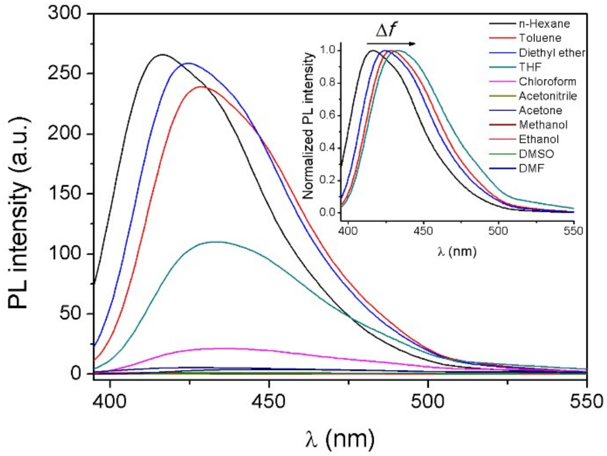

Figure S8. Fluorescence emission spectra of **AAPP-CF3** in different solvents. Inset: Normalized fluorescence emission spectra of **AAPP-CF3** in the solvents with low and medium polarity (from left to right: n-hexane, diethyl ether, toluene, THF). Conditions: The concentration of **AAPP-CF3** was 10 μmol/L. The excitation wavelength was 326 nm.

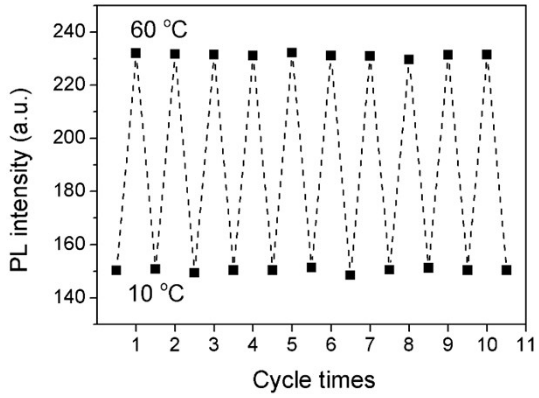

Figure S9. Fatigue resistance of the fluorescence intensity of **AAPP** during 10 heating/cooling cycles in THF. Excitation and emission was performed at 322 nm and 488 nm, respectively.

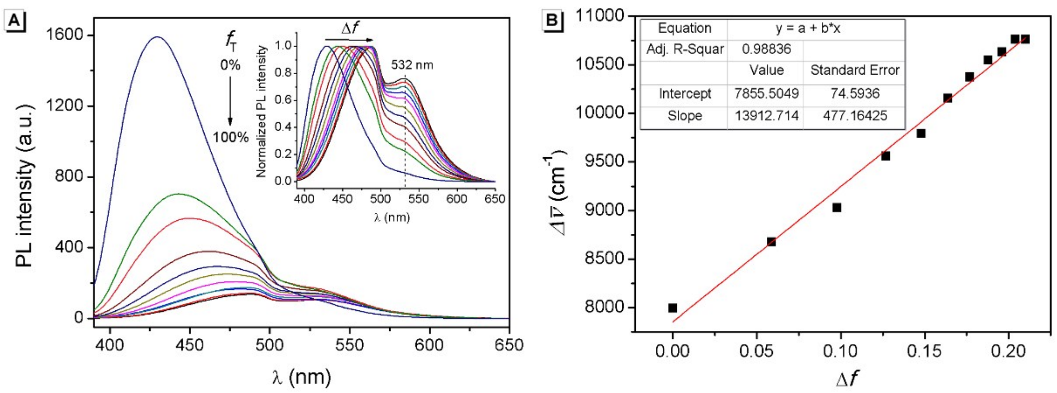

Figure S10. A) Fluorescence emission spectra of **CAPP** in THF/n-hexane with different f_T. Inset: Normalized fluorescence emission spectra. B) Lippert-Mataga plot of **CAPP**. Conditions: The concentration of **CAPP** was 10 μmol/L. The excitation wavelength was 322 nm.

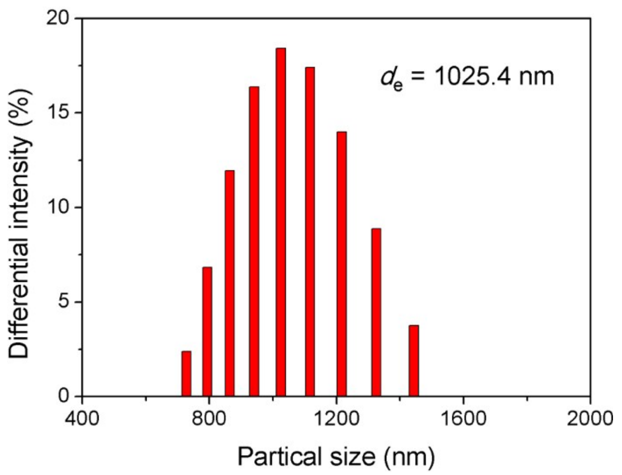

Figure S11. DLS results of **CAPP** in the presence of Cd(II) in aqueous solution of 90% water/THF (*v*/*v*). Conditions: The concentrations of **CAPP** and Cd(II) were 10 μmol/L.

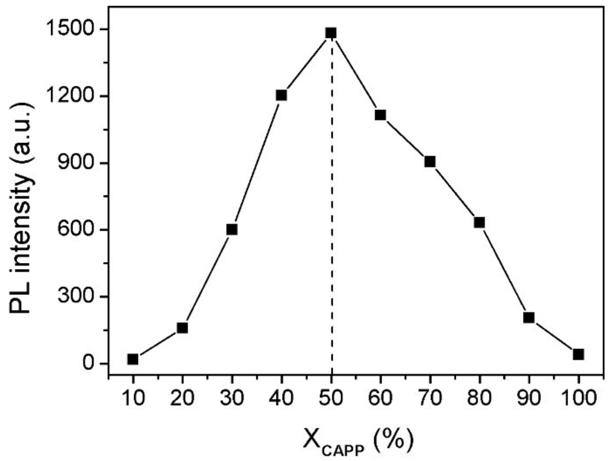

Figure S12. Job's plot data for evaluating the stoichiometry of **CAPP-Cd** complex. $X_{\mathbf{CAPP}}$ was the mole fraction of **CAPP**. Conditions: The total concentration of **CAPP** and Cd(II) was kept at 20 μmol/L. 90% water/THF (*v*/*v*) at pH 7.0 controlled by 10 mmol/L Tris buffer solution. Excitation and emission was performed at 360 nm and 445 nm, respectively.

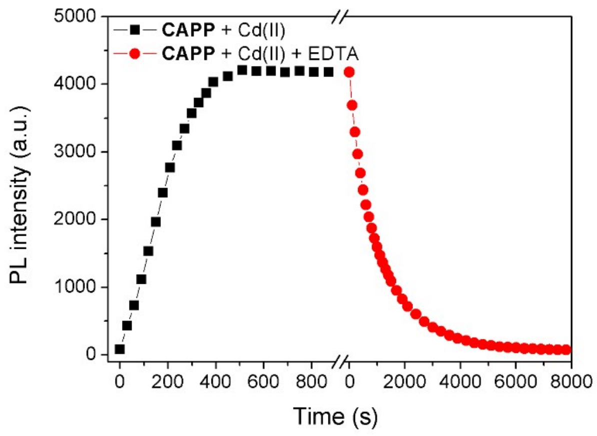

Figure S13. Time-dependent fluorescence spectra of 10 μmol/L **CAPP** with 1 equiv. Cd(II) and 10 equiv. EDTA. Conditions: 90% water/THF (*v*/*v*) at pH 7.0 controlled by 10 mmol/L Tris buffer solution. Excitation and emission was performed at 360 nm and 445 nm, respectively.

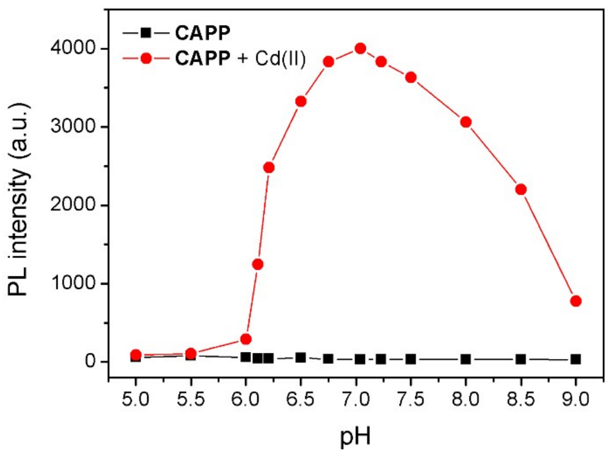

Figure S14. Fluorescence intensity at 445 nm of 10 μmol/L **CAPP** in the absence and presence of 1 equiv. Cd(II) at different pH. Conditions: 90% water/THF (*v*/*v*) controlled by 10 mmol/L Tris and 10 mmol/L HAc/NaAc buffer solutions. Excitation and emission was performed at 360 nm and 445 nm, respectively.

Table S1. Absorption maxima (λ_{abs}^{max}), fluorescence maxima (λ_{em}^{max}), Stokes shift ($\Delta\bar{\upsilon}$), quantum yield (φ), fluorescence lifetime (τ), radiative decay rate constant (k_r) and non-radiative decay rate constant (k_{nr}) of **AAPP** in mixtures of THF/n-hexane with different THF fraction (f_T).

f_T (%)	Δf	λ_{abs}^{max} (nm)	λ_{em}^{max} (nm)	$\Delta\bar{\upsilon}$ (cm^{-1})	φ (%)	τ(ns)	k_r (s^{-1})	k_{nr} (s^{-1})
0	0	319	424	7763	26.68	2.59	1.03×10^8	2.83×10^8
10	0.059	320	442	8626	9.34	3.15	2.96×10^7	2.88×10^8
20	0.098	320	457	9368	4.20	3.11	1.35×10^7	3.08×10^8
30	0.127	320	468	9882	3.00	3.04	9.87×10^6	3.19×10^8
40	0.148	321	476	10144	2.27	2.98	7.62×10^6	3.28×10^8
50	0.164	321	481	10363	1.93	2.91	6.62×10^6	3.36×10^8
60	0.177	321	484	10491	1.68	2.81	5.98×10^6	3.50×10^8
70	0.188	321	486	10577	1.46	2.90	5.03×10^6	3.40×10^8
80	0.196	321	487	10619	1.47	2.93	5.02×10^6	3.36×10^8
90	0.204	322	488	10564	1.41	2.89	4.88×10^6	3.41×10^8
100	0.210	322	488	10564	1.24	2.94	4.22×10^6	3.36×10^8

Table S2. Absorption maxima (λ_{abs}^{max}), fluorescence maxima (λ_{em}^{max}), Stokes shift ($\Delta\bar{\upsilon}$), quantum yield (φ), fluorescence lifetime (τ), radiative decay rate constant (k_r) and non-radiative decay rate constant (k_{nr}) of **AAPP-CF3** in mixed solvents of THF/n-hexane with different THF fraction (f_T).

f_T (%)	Δf	λ_{abs}^{max} (nm)	λ_{em}^{max} (nm)	$\Delta\bar{\upsilon}$ (cm^{-1})	φ (%)	τ(ns)	k_r (s^{-1})	k_{nr} (s^{-1})
0	0.210	368	417	3119	77.95	1.21	6.46×10^8	1.83×10^8
10	0.204	368	420	3218	77.80	1.43	5.42×10^8	1.55×10^8
20	0.196	370	423	3314	70.29	1.71	4.12×10^8	1.74×10^8
30	0.188	371	425	3425	65.10	2.14	3.04×10^8	1.63×10^8
40	0.177	372	426	3480	57.16	2.52	2.27×10^8	1.70×10^8
50	0.164	373	427	3463	47.72	2.79	1.71×10^8	1.87×10^8
60	0.148	373	428	3517	42.23	2.97	1.42×10^8	1.94×10^8
70	0.127	374	429	3572	35.89	3.22	1.11×10^8	1.99×10^8
80	0.098	374	430	3626	31.94	3.44	9.29×10^7	1.98×10^8
90	0.059	374	430	3626	28.64	3.52	8.15×10^7	2.03×10^8
100	0	375	430	3626	26.41	3.59	7.36×10^7	2.05×10^8

4. NMR spectra and HRMS spectra

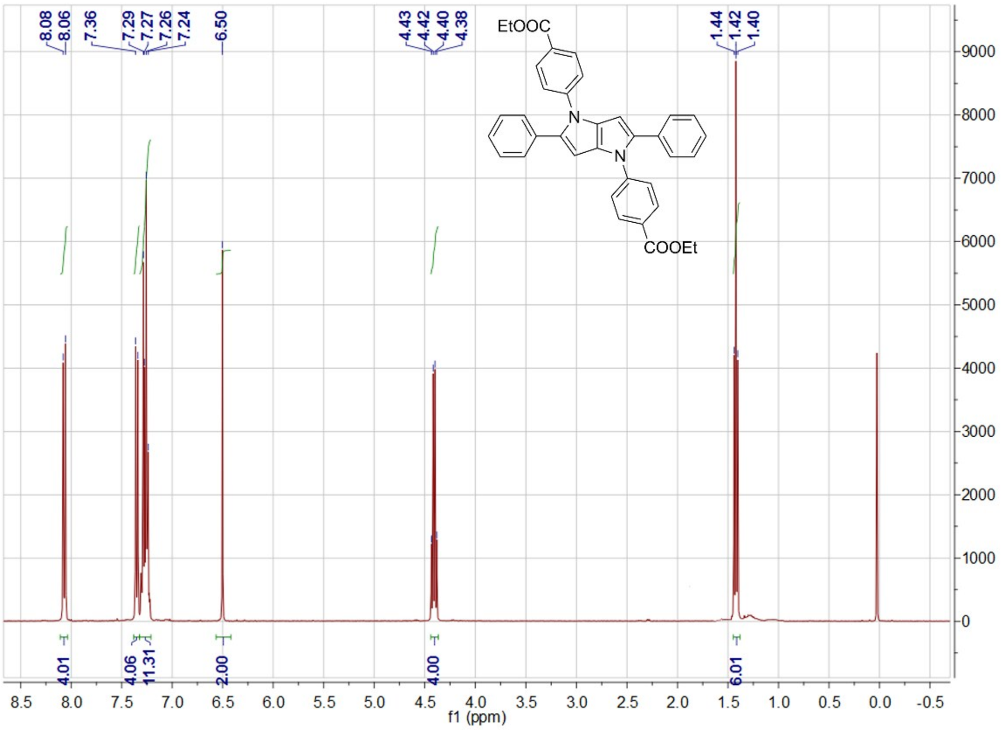

Figure S15. ^{1}H-NMR spectrum of **AAPP**.

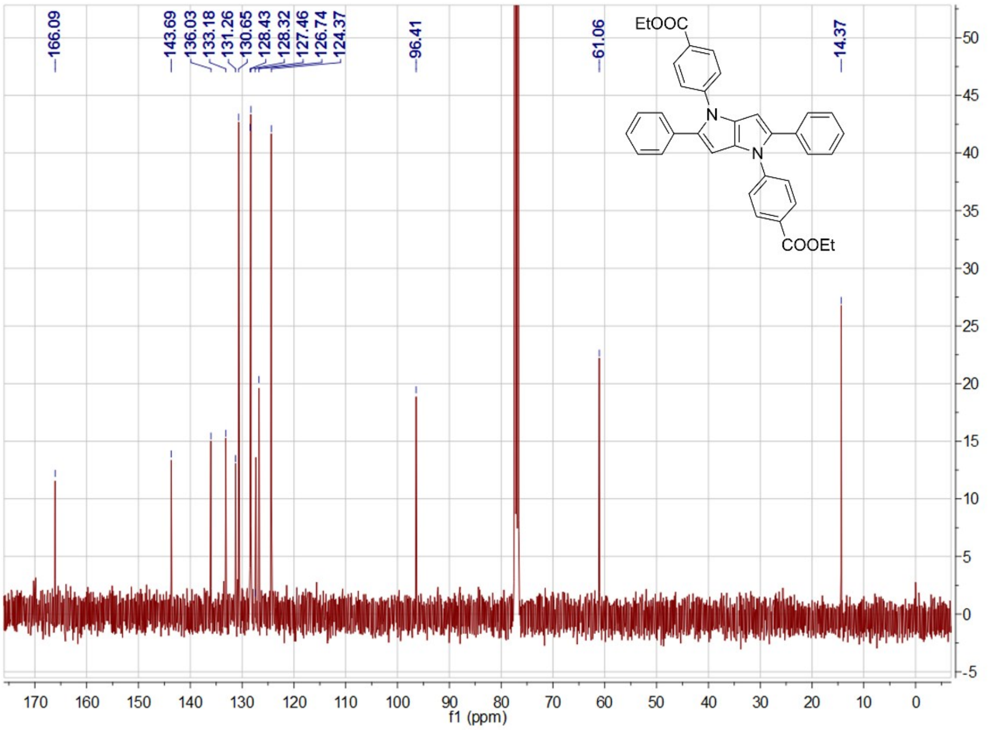

Figure S16. ^{13}C-NMR spectrum of **AAPP**.

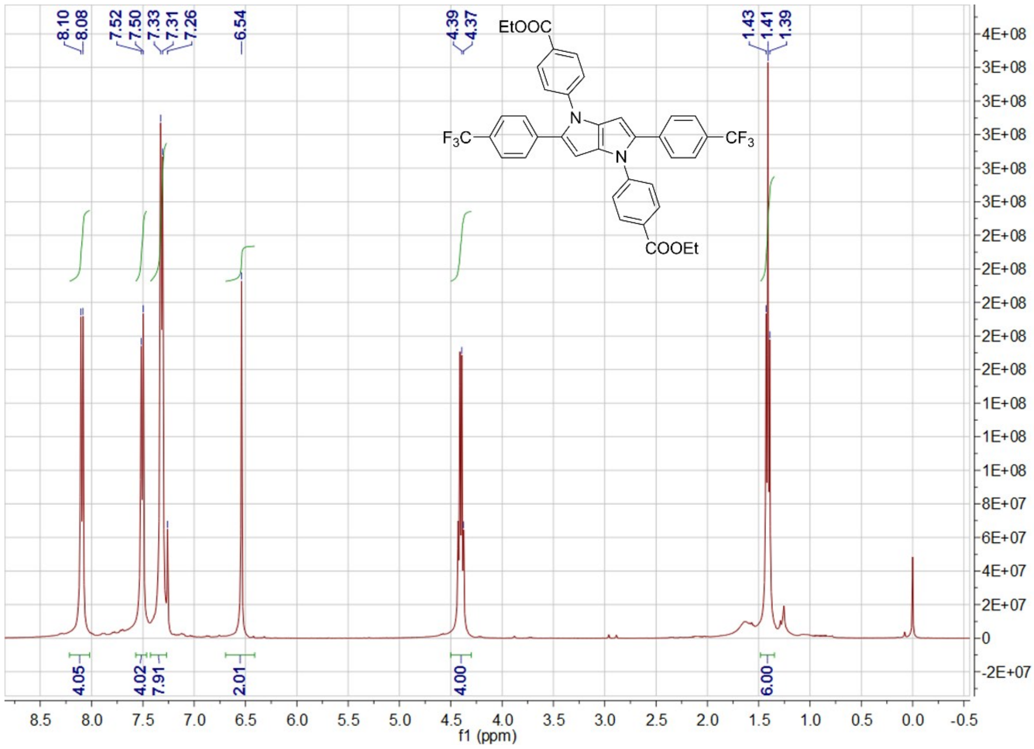

Figure S17. ^{1}H-NMR spectrum of **AAPP-CF3**.

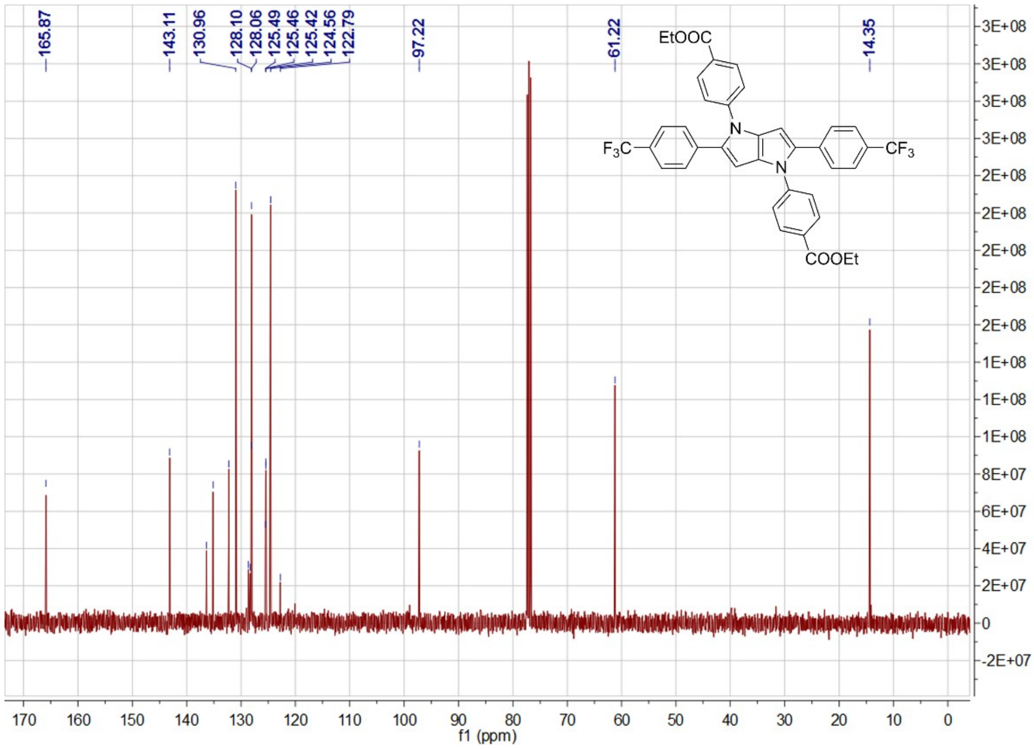

Figure S18. ^{13}C-NMR spectrum of **AAPP-CF3**.

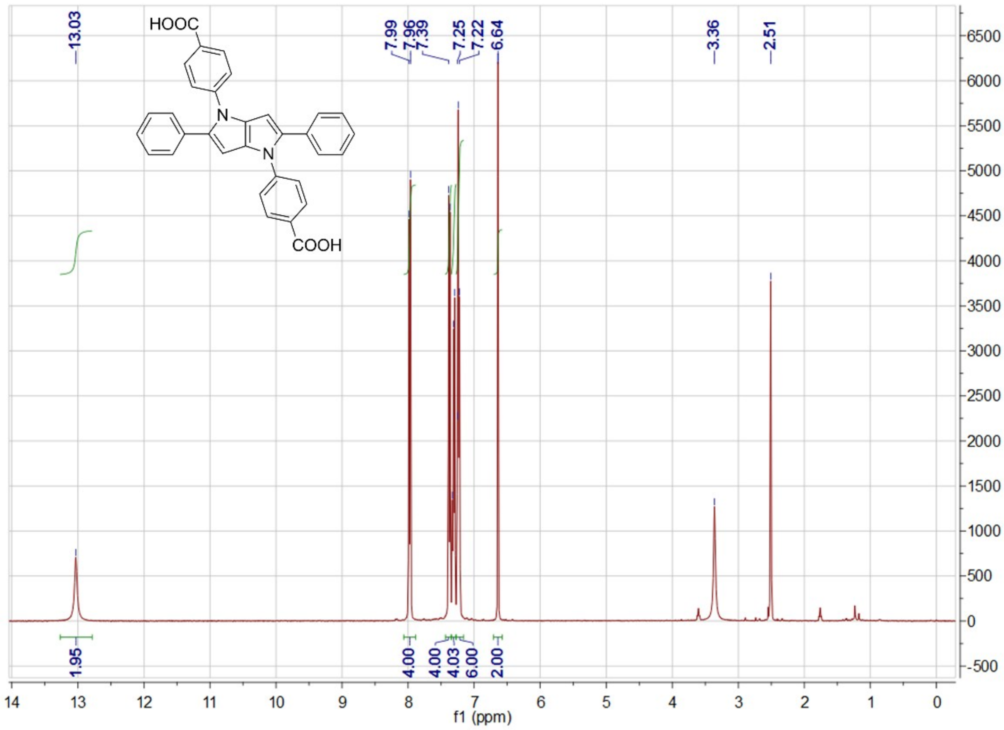

Figure S19. ^{1}H-NMR spectrum of **CAPP**.

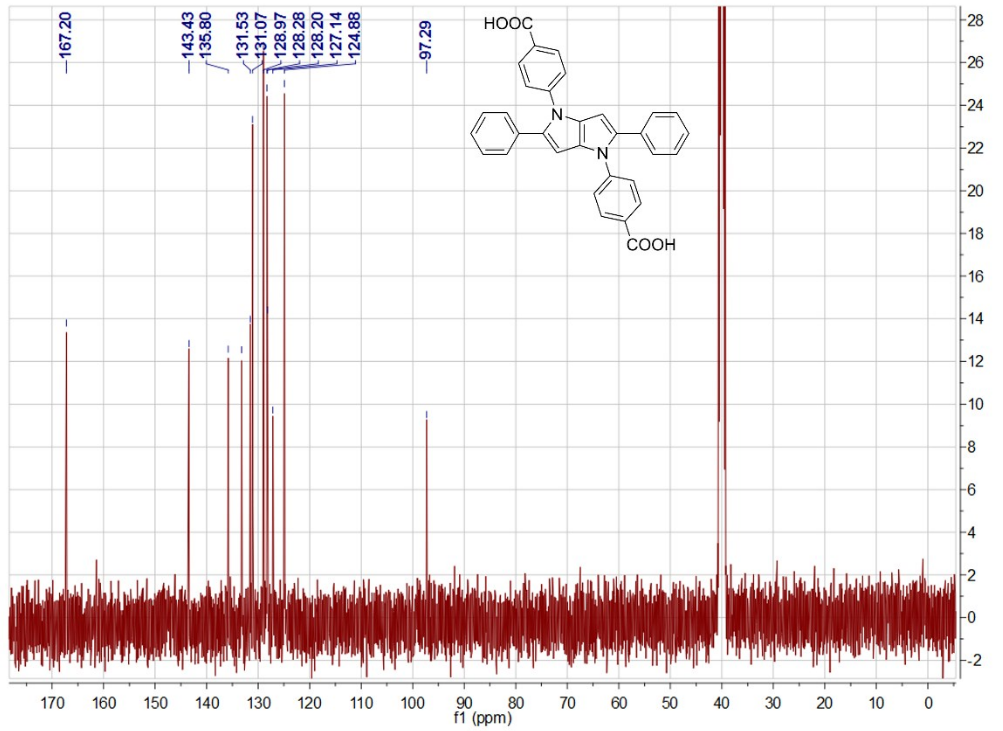

Figure S20. ^{13}C-NMR spectrum of **CAPP**.

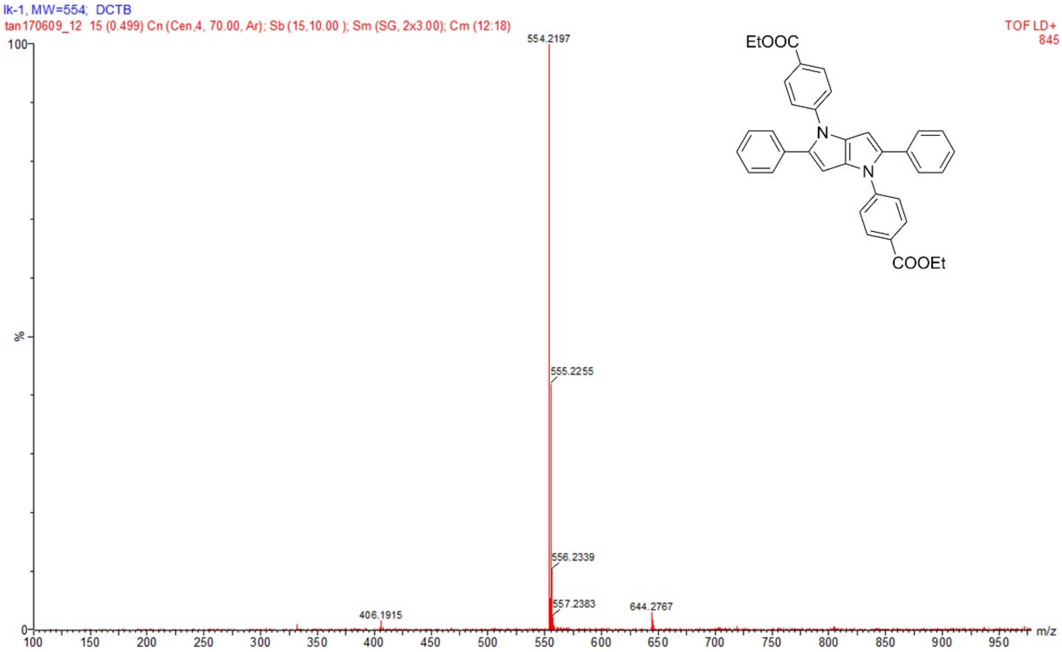

Figure S21. HRMS spectrum of **AAPP**.

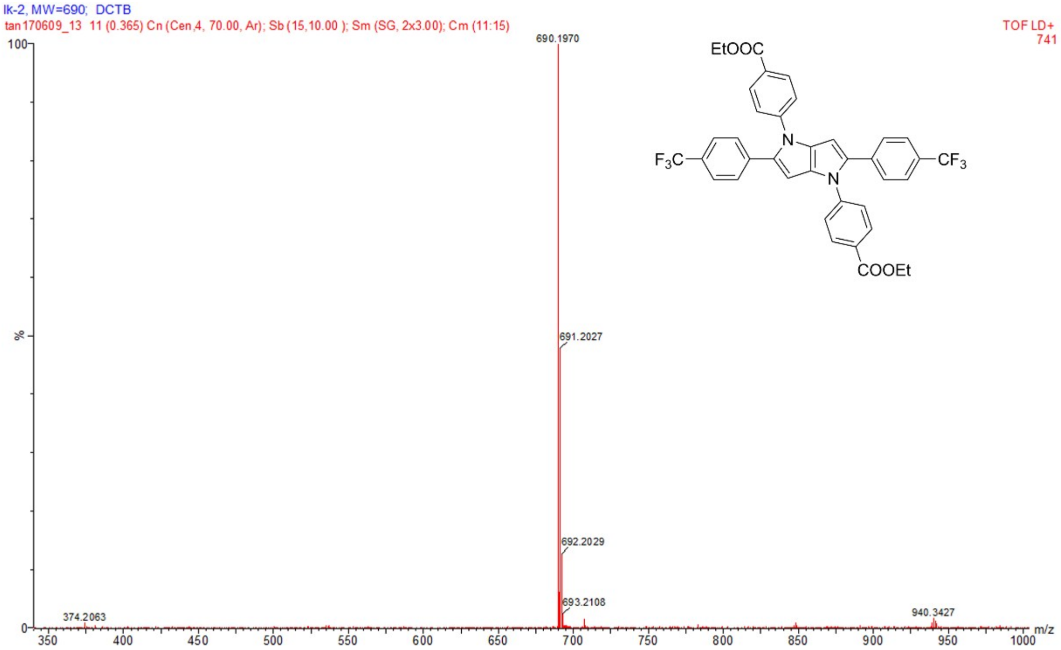

Figure S22. HRMS spectrum of **AAPP-CF3**.

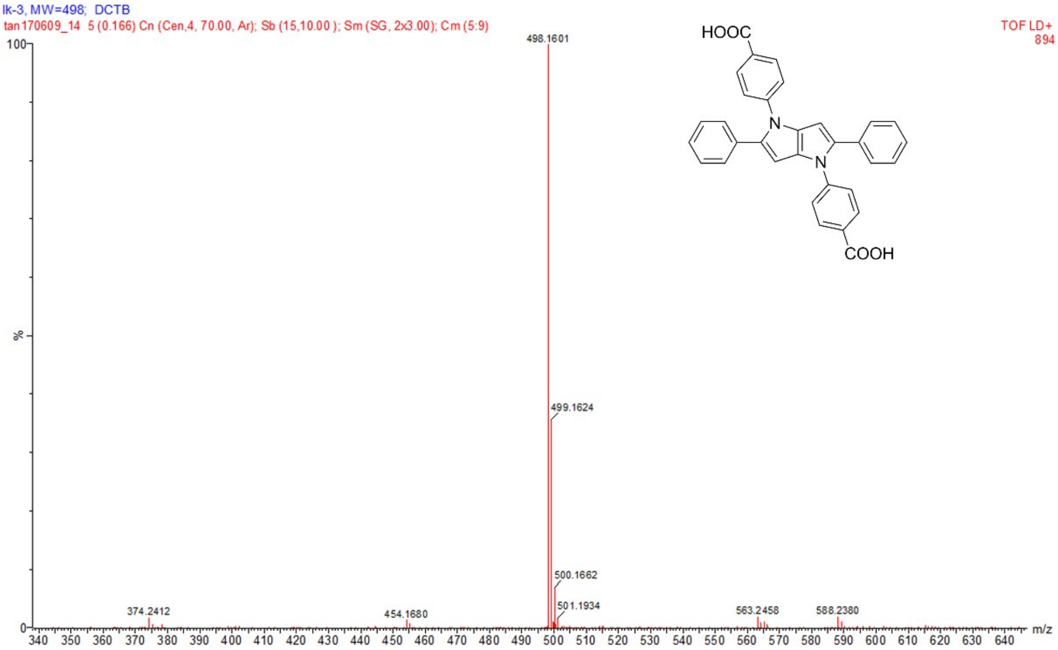

Figure S23. HRMS spectrum of **CAPP**.

5. Crystallographic data and structure refinement

Compound	**AAPP**	**AAPP-CF_3**
formula	$C_{36}H_{30}N_2O_4$	$C_{38}H_{28}F_6N_2O_4$
F_w	554.63	690.63
T/K	293(2)	285.00(10)
Crystsyst	*triclinic*	*triclinic*
Wavelength /Å	0.71073	1.54184
Space group	$P\bar{1}$	$P\bar{1}$
a/Åc	6.1284(12)	6.1875(3)
b/Å	10.938(2)	12.1177(5)
c/Å	11.925(2)	12.2490(5)
α (deg)	114.20(3)	63.176(4)
β (deg)	90.94(3)	88.118(4)
γ (deg)	91.43(3)	81.174(4)
V ($Å^3$)	728.6(3)	809.19(7)
Z	2	2
D_{calcd}(g cm^{-3})	1.264	1.417
F(000)	292.0	356.0
μ (mm^{-1})	0.083	0.987
GOF	1.239	1.055
R_1 (I > 2σ(I))	0.0967	0.0624
wR_2 (I > 2σ(I))	0.2441	0.1758
